# Exploring structure, microbiota, and metagenome functions of epigean and hypogean black deposits by microscopic, molecular and bioinformatic approaches

**DOI:** 10.1038/s41598-022-24159-9

**Published:** 2022-11-12

**Authors:** Beatrice Farda, Ilaria Vaccarelli, Claudia Ercole, Rihab Djebaili, Maddalena Del Gallo, Marika Pellegrini

**Affiliations:** Department of Life, Health and Environmental Sciences, University of L’Aquila, L’Aquila, Italy

**Keywords:** Microbiology, Environmental sciences

## Abstract

This study revealed how Bacteria and Archaea communities and their metabolic functions differed between two groups of black deposits identified in gorge and cave environments. Scanning electron microscopy coupled with energy dispersive spectroscopy was used to analyse the presence of microbial biosignatures and the elemental composition of samples. Metabarcoding of the V3–V4 regions of 16S rRNA was used to investigate Bacteria and Archaea communities. Based on 16S rRNA sequencing results, PICRUSt software was used to predict metagenome functions. Micrographs showed that samples presented microbial biosignatures and microanalyses highlighted Mn concretions and layers on Al-Si surfaces. The 16S rRNA metabarcoding alpha-diversity metrics showed similar Simpson's and Shannon indices and different values of the Chao-1 index. The amplicon sequence variants (ASVs) analysis at the different taxonomic levels showed a diverse genera composition. However, the communities of all samples shared the presence of uncultured ASVs belonging to the Gemmatales family (Phylogenesis: Gemmataceae; Planctomycetes; Planctomycetota; Bacteria). The predicted metagenome functions analysis revealed diverse metabolic profiles of the Cave and Gorge groups. Genes coding for essential Mn metabolism were present in all samples. Overall, the findings on structure, microbiota, and predicted metagenome functions showed a similar microbial contribution to epigean and hypogean black deposits Mn metabolism.

## Introduction

Manganese is the second-most abundant transition metal available on the terrestrial surface and covers 0.1% of the Earth's total mass. It represents a crucial trace nutrient for the growth and survival of numerous living organisms, contributing, for instance, to oxygen production, redox reactions, or protecting cells from toxic metals, UV radiation and oxidative stress^1^. The manganese oxides control the availability of several elements because of their strong oxidizing capacity. The geochemical behaviour of Mn differs based on the oxygen profile^2^. Even if Mn occurs in seven different oxidation states (from 0 to + 7), manganese interacts as reduced soluble or adsorbed Mn^2+^, insoluble Mn^3+^ and Mn^4+^ oxides in the natural environment^3^. Direct and indirect evidence for the biogenic Mn coatings, such as crusts, and nodules, can occur in various environments on the Earth's surface and subsurface^3^. Typical natural ecosystems hosting nodules, black crusts, mineralized stromatolites, patinas, and rock varnishes of manganese are deserts, seawater, freshwaters, and caves^3^. The frequent occurrence of these intriguing formations in different environments has also been described by Darwin during his iconic voyage aboard the HMS Beagle. However, their origins are still partially unknown, with both the biogenic and abiotic models demonstrated in nature. When the conditions are favourable, different microbial groups can control the oxidation of Mn, competing with the abiotic process^4^.

Mn and other metals, play a major role in controlling both the redox balance and the carbon cycling via oxidation in the environments in which these elements are abundant^5^. Microorganisms, mainly bacteria and fungi, can perform oxidation/reduction of Mn for carbon oxidation and growth^6^. Mn microbial redox reactions of Mn(II) to Mn(IV) are most common, with Mn(III) as an occasional intermediate. In many cases, coupling Mn(IV) reduction to the complete oxidation of organic matter is reported^5^. Mn-oxidizing bacteria harbour a suite of enzymes that help scavenge Mn and other associated elements, while several Mn-reducing microorganisms, from highly aerobic to strictly anaerobic, have been described in the literature^7^. The microbial mechanisms of Mn reduction can be either an indirect process resulting from interactions with organic or inorganic compounds or comprising natural enzymatic processes based on an electron-transfer reactions^6^.

Oxide minerals associated with a biogenetic formation of black deposits are birnessite and todorokite, frequently found within rock varnish and ocean nodules and in black deposits from caves^7^. Specifically, Spilde et al. compared these two oxide minerals from cave deposits with those found in rock varnish and described a progression of increasing crystallinity from a filamentous to a fibrous form for those found underground^8^. From a morphologic point of view, Manganese oxides found in caves are different from the oxides in rock varnish. The manganese oxides from rock varnish are undulating, sometimes discontinuous, and laminated with clay and quartz, most likely dust particles. On the other hand, the cave coatings are thick and with a softer consistency^9^. Spilde suggests that the laminae that occur in rock varnish are wetted and dried because they were subjected to harsh conditions resulting in thin, flat layers, while in caves, the rock layers tend to preserve black crusts, shielded from the corrosion by weathering. Although there are these micro-and macroscopic differences, Spilde found similarities in the microbial communities between the cave and rock surface coatings^8^.

Studying microbial contribution to minerals formation can be challenging. Generally, deposits harbour microbial biosignatures that can help speculate about the microbial presence. However, preserving well-defined microbial structures is difficult because the cells collapse and degrade. In addition, the manganese oxides degrade the organic compounds left behind by living cells, and thus, they leave only a few traces of biological activity^10^. Mn oxidation/reduction mechanisms are still under debate, and more experimental research is necessary to understand the enzymatic pathways involved. The RNA-seq analysis is the most powerful tool to gather the differentially expressed genes within a community. However, few attempts to sequence ancient transcriptomes were carried out due to the high-degradative nature of RNA. The 16 s rRNA metabarcoding and bioinformatic tools may help obtain helpful information about the prokaryotic community (mainly *Bacteria* and *Archaea*) and metagenome functions involved^11–14^.

In our previous studies we investigated and described black deposits of gorge and cave environments, finding similar biological and structural traits^12,15^. For this reason, we hypothesized that black deposits of gorge and cave environments could present similar Bacteria and Archaea communities and common metabolic processes, especially concerning manganese metabolism. This work aims at investigating similarities and differences among the deposits of these two different environments, the similarities and differences among Bacteria and Archaea communities and metagenome functions that these hypogean and epigean environments share between two groups of black deposits found in gorge and cave environments. To evaluate the presence of microbial biosignatures and the element composition, samples were investigated by scanning electron microscopy coupled to energy dispersive spectroscopy (SEM–EDS). The Bacteria and Archaea communities were investigated by metabarcoding of V3-V4 regions of 16S rRNA. To predict the metagenome functions, sequencing data were analysed by PICRUSt (Phylogenetic Investigation of Communities by Reconstruction of Unobserved states, 2) software^11^.

## Results

**Figure 1:**
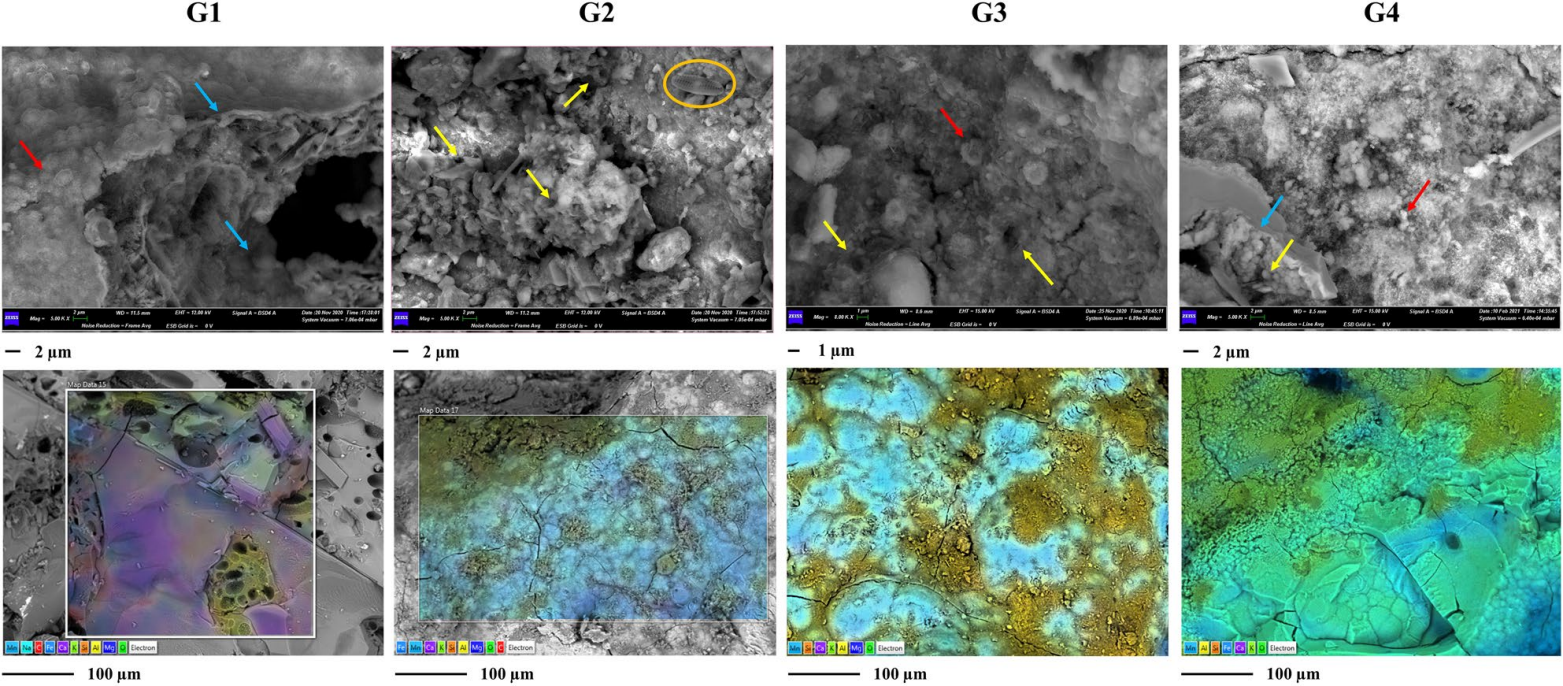
SEM–EDS investigations of gorge samples. Upper panels show the micrographs obtained by SEM observations. In the panels the putative microbial imprints are marked by yellow arrows, the microbial biofilms by blue arrows, and microbial mineralized structures by red arrows. The orange circle in sample G2 marks the presence of a diatom within the sample. Work distance (WD) = 8.5–11.5 mm; electron high tension (EHT) = 12–15 kV; Signal = BSD4 A (backscattered electrons); Magnitude = 5–8 K X (Scale bars are presented under each panel). Lower panels present the elemental maps obtained by EDS analyses. The microanalysis acquisitions were carried out at 12.00 kV (Scale bars are presented under each panel). Mn presence is underlined with light blue colour.

**Figure 2:**
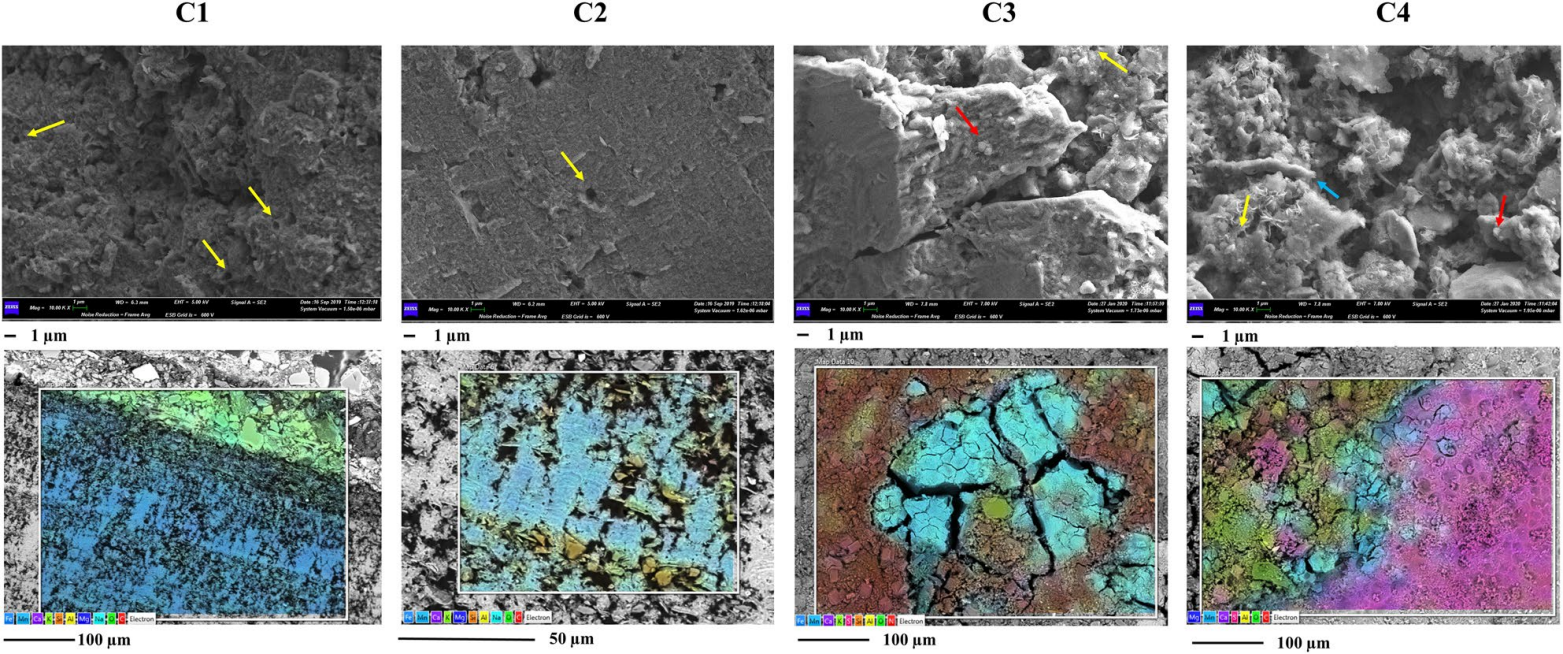
SEM–EDS investigations of cave samples. Upper panels show the micrographs obtained by SEM observations. In the panels the putative microbial imprints are marked by yellow arrows, the microbial biofilms by blue arrows, and microbial mineralized structures by red arrows. Work distance (WD) = 6.2–7.8 mm; electron high tension (EHT) = 5–7 kV; Signal = SE 2 (secondary electrons); Magnitude = 10 K X (Scale bars are presented under each panel). Lower panels present the elemental maps obtained by EDS analyses. The microanalysis acquisitions were carried out at 12.00 kV (Scale bars are presented under each panel). Mn presence is underlined with light blue colour.

The black deposits were investigated by SEM–EDS. As presented in Figs. 1 and 2, both Gorge’s and Cave’s samples showed the presence of multi-layered structures and filaments attributable to microbial aggregation structures.

**Figure 3:**
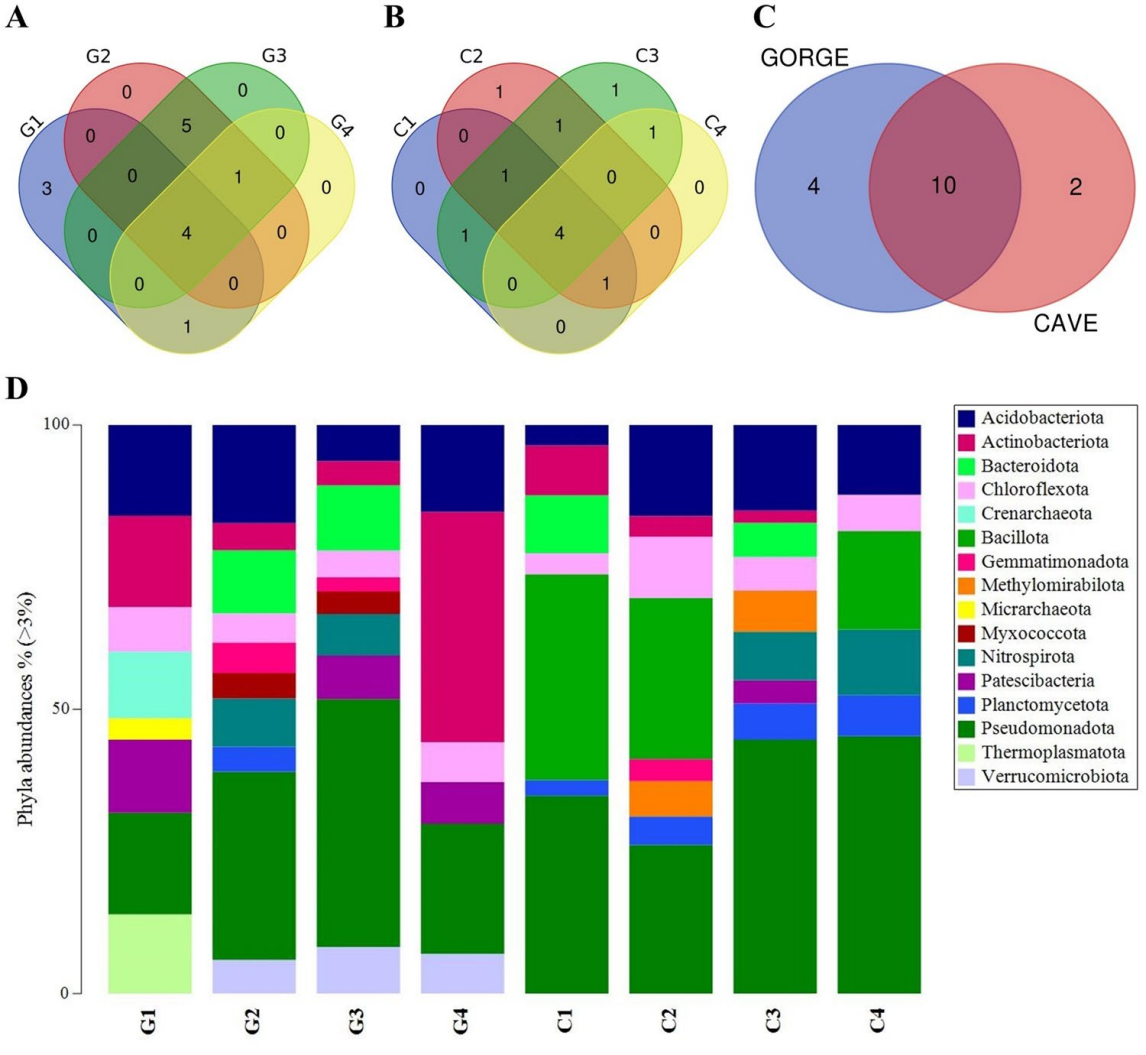
Venn diagrams and taxonomy barplot of ASVs at phylum level. Venn diagrams show common phyla found within gorge (**A**) and cave (**B**) samples and between the two groups (gorge vs cave). Taxonomy barplot shows the abundances (%) of main phyla in each sample (**D**) (cutoff 3%).

The multi-layered structures were characterized by biofilms leaning on layers of mineral surfaces. Within these layers, many bores and cell-like shapes were found. Gorge's samples also showed the presence of many fossil diatoms embedded in mineral structures and concretions (Sample G2, Fig. 3). Figures 1 and 2 (Supplementary Figs. S1 and S2) also show the elemental composition of the samples. Mn layers were deposited or alternated with mineral fragments. The observations suggest the presence of a basic Si-Al mineral surface, with the same distribution and a distinct distribution to the other elements. This Si-Al mineral surface presented covering layers and concretions of Mn in all samples. The diffuse presence of other elements was also shown (i.e., Fe, K, Mg, and Na). In some cases, the distinct spatial distribution of Mn to S (Samples C3 and C4) and strict association with Fe (Sample C4) were observed. The Ca element was more diffuse in the cave's samples. Layers and concretions were instead observed in the gorge’s samples.

The black deposits of the Gorge and Cave groups were subjected to 16S sequencing. The ecological indices obtained for the analysed samples are shown in Table 1. Sample abundances ranged from 461 to 2045 (Chao-1), with samples G1 and C1 having the lowest values and C2 and C3 having the highest. The number of individuals displayed was comparable to the abundance recorded, implying a satisfactory sampling method. All the samples had substantial levels of diversity (H’ values higher than 4.5 and S 1-D near 1). The sample G2 had the most diversity, while the sample G1 had the least.

**Table 1:** Alpha-diversity metrics obtained for gorge (G1-G4) and cave (C1-C4) samples.

	G1	G2	G3	G4	C1	C2	C3	C4
Number of reads	28,169	21,297	35,246	24,417	52,407	55,588	25,207	41,352
Taxa_S	460	1807	1197	1407	806	2115	2043	1105
Chao-1	461	1814	1200	1413	822	2119	2045	1116
Shannon_H	4.7	7.0	6.4	6.6	5.2	6.5	6.4	5.9
Simpson_1-D	0.977	0.998	0.997	0.997	0.980	0.992	0.992	0.988

Sequencing results with a cutoff of 3% were processed and analysed at Domain, Phylum, Class, and Genus levels. At the Domain level, except for sample G1, which showed 27% Archaea and 73% Bacteria, the taxonomic data processing revealed that Bacteria accounted for most of the samples (97–100%) (Supplementary Table S1). The study of the total number of different ASVs belonging to each phylum (Supplementary Table S2) showed that the highest number of different ASVs was found in C2 for Acidobacteriota and Pseudomonadota and in G2 for Chloroflexota. The lowest values were recorded for G1. No marked differences were found among samples based on environment type. Except for sample G1, which included only Crenarchaeota, Micrarchaeota, and Thermoplasmatota, the Gorges samples were quite similar in their composition at the phylum level (Fig. 3A; Supplementary Table S2). For Cave samples, the situation was similar, with Gemmatimonadota and Patescibacteria being only present in samples C2 and C3, respectively (Fig. 3B; Supplementary Table S3). Nitrospirota, Actinobacteriota, Pseudomonadota, Chloroflexi, Patescibacteria, Acidobacteriota, Planctomycetota, Bacteroidota, and Gemmatimonadota were among the nine phyla that the Gorge and Cave groups shared (Fig. 3C; Supplementary Table S3). The Pseudomonadota phylum had a comparable abundance in all the samples, as shown in Fig. 3D. The other common phyla were present with different abundances. Therefore, at the class and genus levels, the differences between Gorge and Cave groups were more evident, underlying different compositions and abundances. However, some common traits were still found.

**Figure 4:**
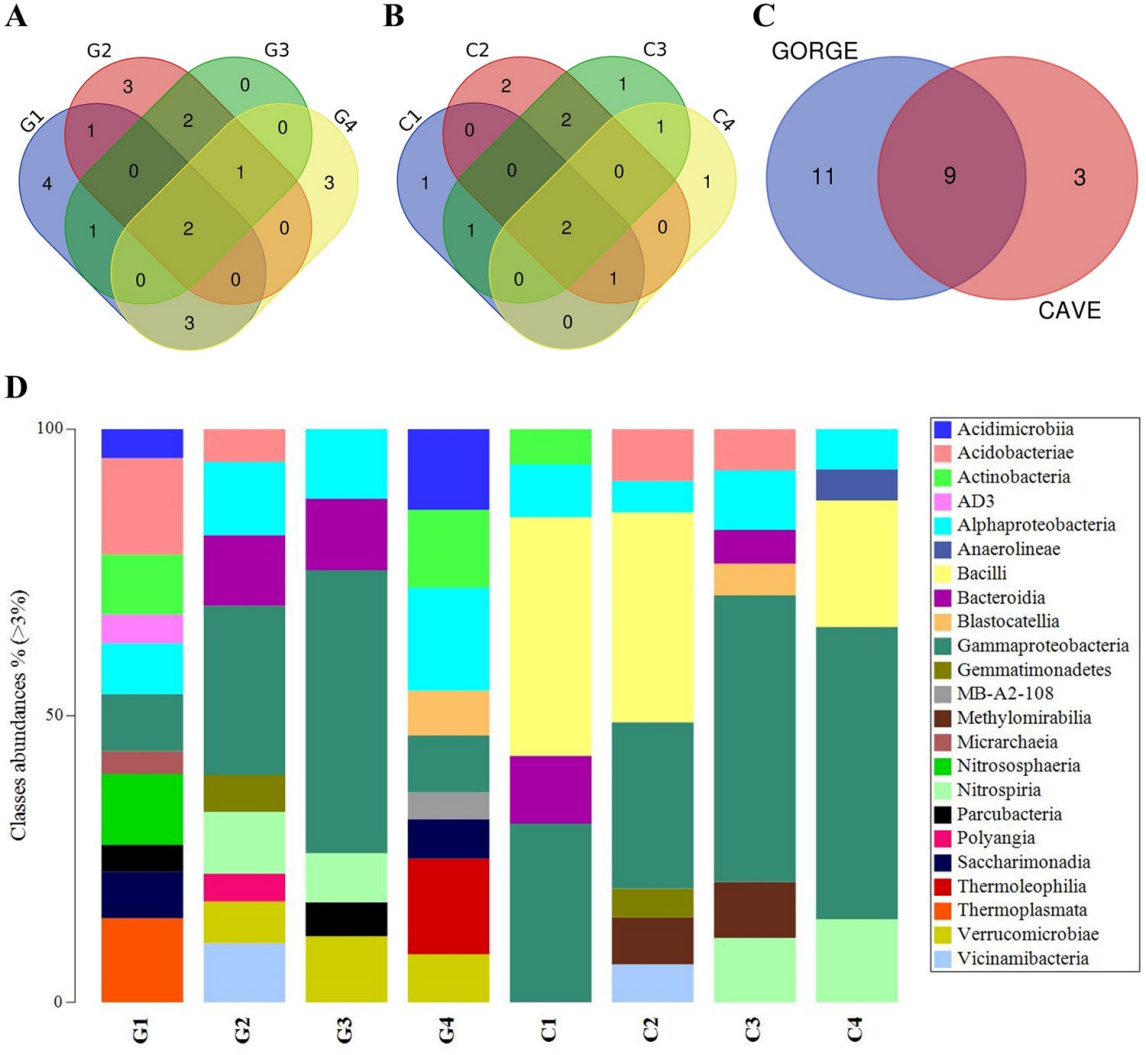
Venn diagrams and taxonomy barplot of ASVs at class level. Venn diagrams show common classes found within gorge (**A**) and cave (**B**) samples and between the two groups (gorge vs cave). Taxonomy barplot shows the abundances (%) of main classes in each sample (**D**) (cutoff 3%).

At the Class level, Alphaproteobacteria and Gammaproteobacteria were present in all samples of Gorge and Caves (Fig. 4A,B, respectively; Supplementary Table S4). Beyond these two classes, Vicinamibacteria, Bacteroidia, Gemmatimonadetes, Actinobacteria, Acidobacteriae, Blastocatellia, and Nitrospiria were found common between the two groups (Fig. 4C; Supplementary Table S4). Alphaproteobacteria and Gammaproteobacteria accounted for similar abundances within the bacterial communities of the samples (Fig. 4D).

**Figure 5:**
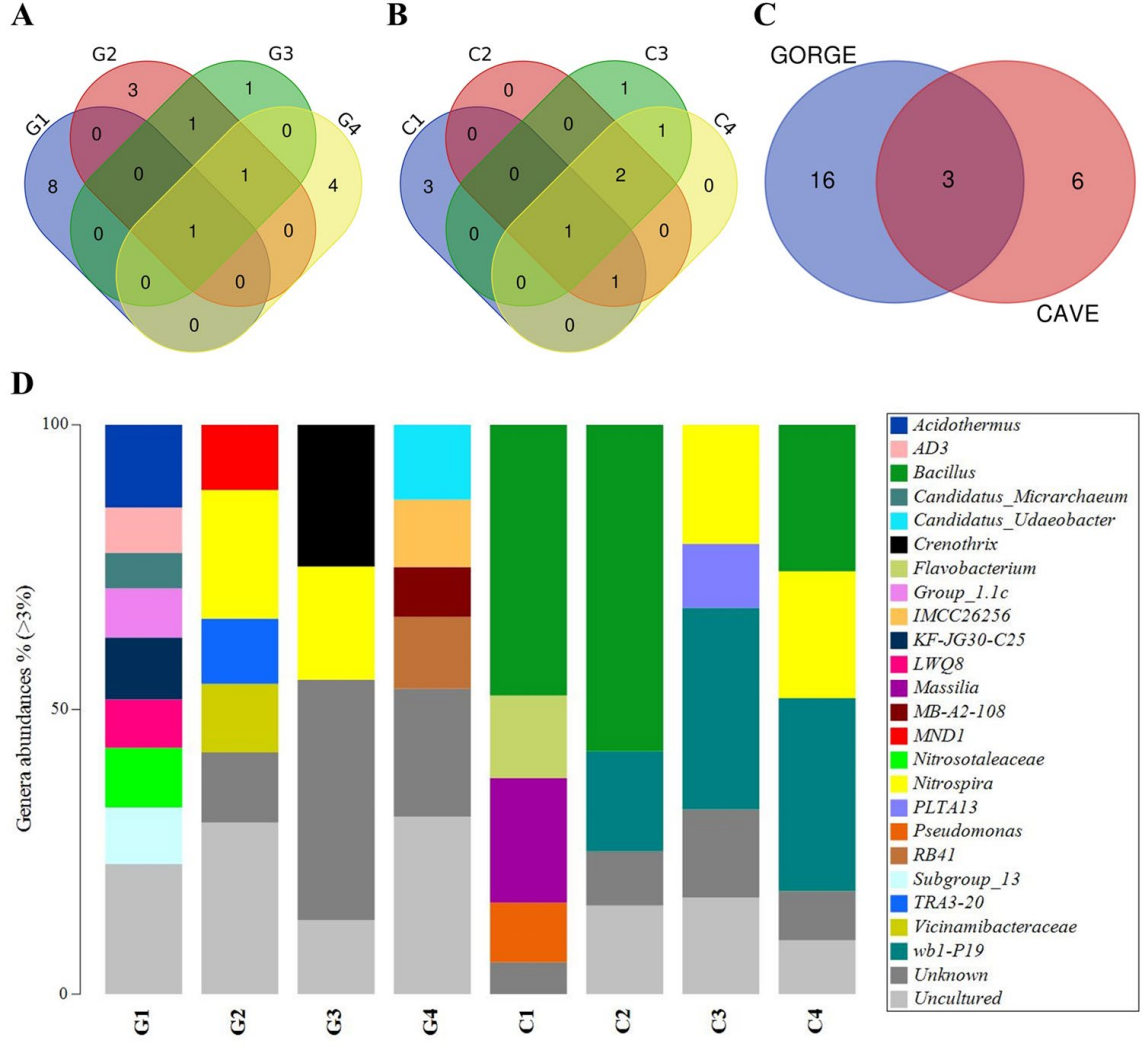
Venn diagrams and taxonomy barplot of ASVs at genus level. Venn diagrams show common genera found within gorge (**A**) and cave (**B**) samples and between the two groups (gorge vs cave). Taxonomy barplot shows the abundances (%) of main genera in each sample (**D**) (cutoff 3%).

Only the uncultured group was common at the Genus level in Gorge and Caves samples, respectively (Fig. 5A,B; Supplementary Table S5). The uncultured group mainly belonged to the Gemmatimonadaceae family (Phylogenesis: Gemmatales; Planctomycetes; Planctomycetota; Bacteria) but was not representative of all the samples (only G1 and G4). Except for sample G1, the unknown group was also common. The ASVs of this group mainly belonged to the Hydrogenedensaceae family (also present in G1 with abundance < 3%). The *Nitrospira* genus was found common in both groups (Fig. 5C; Supplementary Table S5). Except for C1, the uncultured and unknown groups accounted for a relevant abundance in all the samples (Fig. 5D). The other relevant genera were *Acidothermus* (for G1), *Nitrospira* (for G2, G3, C3, and C4), *Crenothrix* (for G3), *Bacillus* (for C1, C2, and C4), and *wb1-P19* (for C2, C3, and C4).

**Figure 6:**
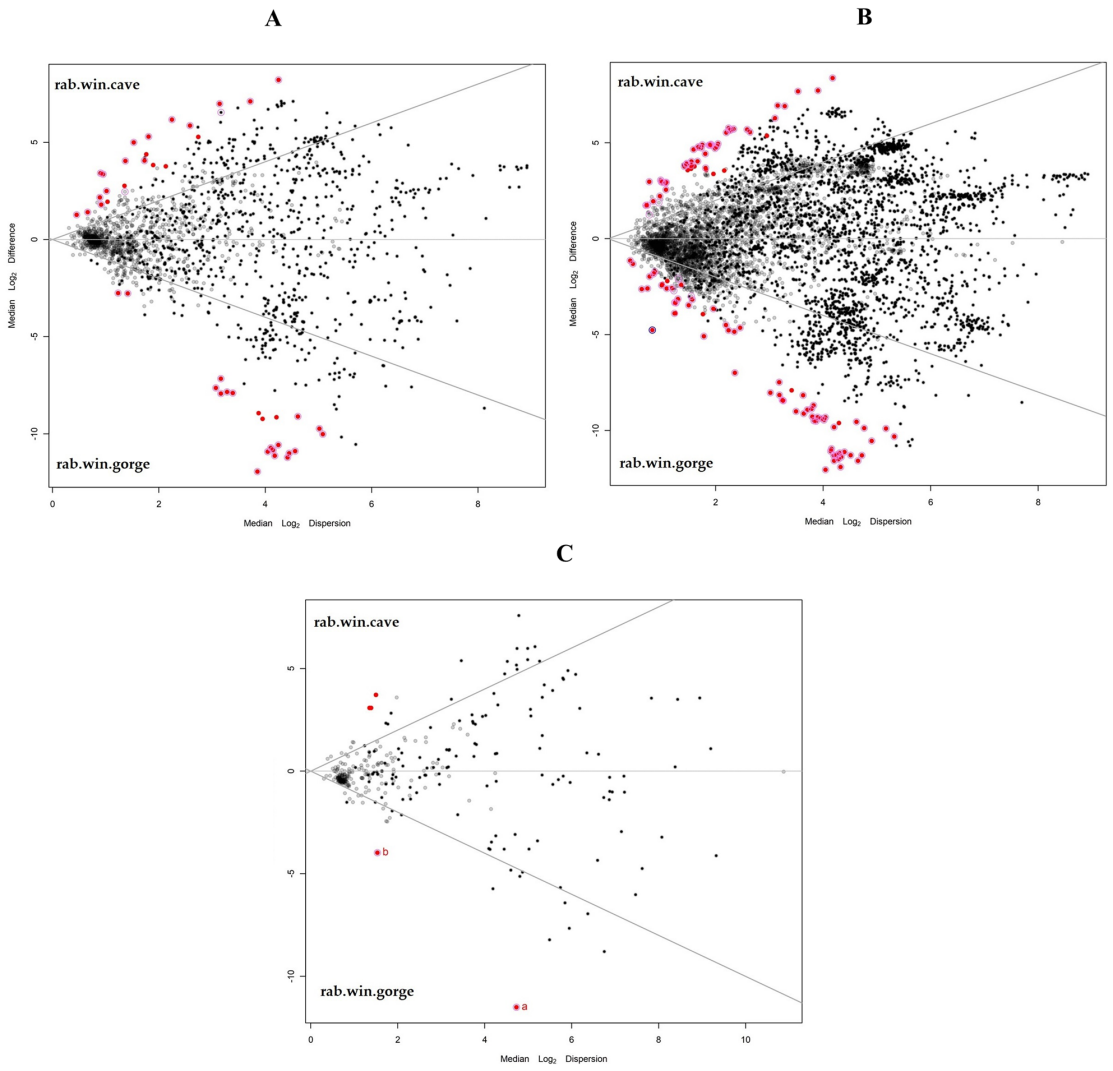
Volcano plots showing the significant differences (red dots) in predicted enzymes (**A**), keg orthology (**B**), and pathways (**C**) by PICURUSt software.

To predict the microbial community functional profiles, the samples were also processed by using PICRUSt software. The EC, KO, and PWY outputs were firstly analysed to investigate similarities among the samples. Supplementary Fig. S3 reports the cluster outputs obtained for EC, KO, and PWY. All the evaluations underlined two distinct clusters based on sample origin. The Cave samples clustered within the first group, while Gorge samples clustered within the second group. To evaluate the differences among the groups, data were further investigated statistically. Figure 6A shows the differences among the EC predicted for each group. The statistical comparison showed that many enzymes were predicted differently based on Gorge and Cave samples. The enzymes with a significant statistical difference belonged to oxidoreductases, transferases, hydrolases, lyases, isomerases, and ligases families (statistical differences shown in Supplementary Table S6). Figure 6B depicts the differences among the KO predicted for each group. The evaluation showed that many predicted KO were statistically different between Gorge and Cave groups. Among them of relevance was the significant abundance within the gorge group of K16080, the functional ortholog of high-affinity Mn^2+^ porin (mnoP) (statistical differences shown in Supplementary Table S7). Figure 6C presents the statistical comparison of the PWY predicted for gorge and cave groups.

**Figure 7:**
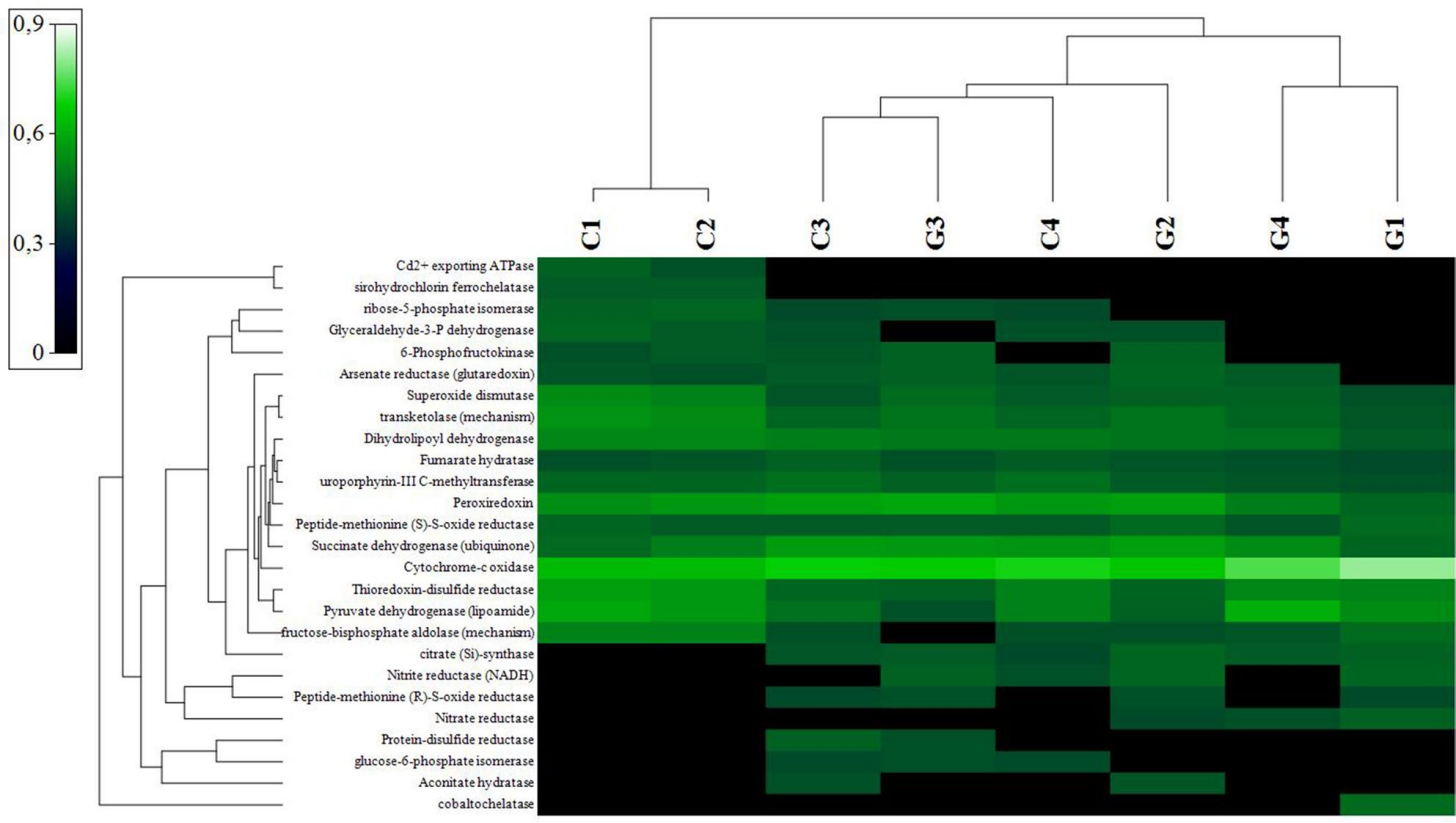
Heat map of the enzymes involved in Mn metabolism predicted by PICURUSt software. The upper clusterization shows the sample distribution according to enzymes presence/absence and abundances. Heat map was realized based on resemblance matrix (Analyse between variables, measuring the index of association) and hierarchical cluster analysis (Group average cluster mode).

The investigation showed that only P621-PWY, nylon-6 oligomer degradation (a) and PWY-6876 (b), isopropanol biosynthesis, were different (Supplementary Table S8). This aspect suggested that even if the samples were constituted by different genera and had different predicted EC and KO, the pathways involved were mostly similar. To find out more about the potential redox biotransformation, metal resistance and respiratory processes, the EC outputs were also checked for the presence of enzymes involved in these processes. Figure 7 shows the heatmap generated for the most relevant abundances. Among the common enzymes, all samples with high abundances showed cytochrome-c oxidase, peroxiredoxin, and superoxide dismutase. The different distribution and abundance of other enzymes, such as Cd^2+^ exporting ATPase and sirohydrochlorin ferrochelatase, allowed clustering of the samples C1 and C2 in a different group. Sample G1 showed the abundance of most of the EC investigated and was the only sample found with cobaltochelase with good abundances.

Data on alpha-diversity indices, phylum, Mn-involved EC, and environmental variables were also used to conduct a principal component analysis (Supplementary Fig. S4 and Supplementary Table S9). The results showed that the samples of gorge and cave groups occupied separated clusters, with temperature accounting more for gorge samples and pH and relative humidity for cave ones. For gorge site the temperature is more variable during the days and the seasons.

## Discussion

Black deposits of gorge and cave environments have been investigated for many years^3,5,8^. However, despite the similarities they present, as far as we know, no studies dealt with the comparison of the Bacteria and Archaea communities for the identification of differences and similarities. The SEM–EDS investigation allowed to underline the presence of microbial imprints within the samples with a marked association of biological elements with the mineral matrices and the presence of Mn oxides layered upon a Si-Al mineral surface. These strict associations of the bacteria signatures within mineralized structures allow speculating on the contribution of microorganisms to the formation of these deposits. Microbial contribution is usually described by the mineralization/lithification of microbial cells and their aggregation structures, such as biofilms^12^, which can occur internally and externally. Many authors have described the presence of these biosignatures for several speleothems^15–18^. Our previous investigations described already the presence of these imprints within “Grotta Grande dei Cervi” black deposits^12,19^. This cave is one of the main caves present in the karst system of Pietrasecca together with “Ovito di Pietrasecca”. However, for the latter, microscopic observations of black deposits have not yet been reported in literature. Even with similar microbial imprints, the samples from “Ovito di Pietrasecca” (C3 and C4) showed a more prominent presence of N and P elements than those from “Grotta Grande dei Cervi”. These aspects should be further investigated by lithological investigations. However, we could speculate that this difference can be associated with different speleogenesis, which is still active in “Ovito di Pietrasecca” and inactive in “Grotta Grande dei Cervi”. These dynamics promote different environmental conditions, with a critical role in nitrogen and phosphate participation in the formation and degradation of primary and secondary deposits.

For gorge samples, the presence of black deposits is within ravine walls at low heights. This suggests that their formation dates to ages ago. As previously presented, this gorge develops primarily on volcanic substrates covering a sedimentary origin. Less than 2 million years ago, the waters of the Pliocene Sea covered all this emerged area. Following the marine regression, the young watercourses' volcanic genesis and erosive action gave rise to the deep incisions. Given the substantial presence of interactions between microbial and mineral imprints, the contribution of bacteria seems to have been influential in the formation of these deposits. Knowledge of these aspects and the observation of sampling sites lead us to associate these deposits with marine crusts-like deposits^20^, for which microbial activity is essential^21^. This speculation was supported by numerous fossils/traces of diatoms highlighted with SEM observations.

The 16S rRNA metabarcoding showed common ASVs at phylum and class levels. At the phylum level, Pseudomonadota was ubiquitous and represented important abundances. Pseudomonadota is one of the phyla that play a crucial role in the biogeochemical cycle of Mn^6,22^. It is represented by many Mn-oxidizing bacteria and is found in various settings (e.g., groundwaters, caves, soil)^23^. Alphaproteobacteria and Gammaproteobacteria are the classes with a joint distribution. For these classes, Mn oxidation is usually described by animal heme peroxidases (AHPs) and the multicopper oxidases (MCOs) enzymes, also found in the predicted metagenome functions of our study^24^. All the samples shared uncultured and unknown groups at the genus level. These groups are present in microbial communities of all types of environments. Uncultured bacteria are a key element of microbial communities. They represent a hidden population that provides an unexploited genetic resource encoding unique and valuable catalysts and enzymes. These aspects are important both for environmental and industrial application aspects^25^.

Among unknown ASVs, the representative taxon was assigned to the Hydrogenedensaceae family, common in all the samples. Limited information is available on the ecological role of this lineage. Their description in different types of wastewater treatments^26,27^ indicates anaerobic metabolism. Their distribution has been described in different environments. Lusa and Bomberg found this taxon in ombrotrophic Boreal bog communities, describing a distribution positively correlated to the presence of mineral elements (i.e., Mg, Al, Fe, Co, Cu, Se, Cs, Th, U) and negatively correlated to the organic matter content^28^. Cohen and collaborators described this family in permanently redox-stratified Fayetteville Green Lake as a part of the particle-associated bacterial community^29^.

Among the significant genera, *Acidothermus* (Actinobacteria phylum) is a thermophilic, acidophilic, and cellulolytic bacterium that can also break down chitin. It was initially isolated from an acidic hot spring in Yellowstone National Park^30^ and has also been described in several ecosystems, including as a part of the oxide sediment bacterial community of the eastern Mediterranean Sea^31^ and of an old Indian pond^32^. *Crenothrix*, are Mn oxidizer bacteria already used in biotechnologies for Mn biomining^33^. *Nitrospira* is the most varied and ubiquitous nitrifying in natural ecosystems and biological wastewater treatment^34^. *Bacillus* is one of the Mn-oxidizing bacteria used as a model due to its extraordinary biomining abilities^35^. Less is known on *wb1-P19.* However, phylogenetic studies suggested sulphur or nitrite-oxidizing autotrophic metabolism^36^ and its presence inside the cave has been already reported^36–39^.

Even with different microbial community structures and compositions, the samples presented similar predicted metagenome functions with some habitat-specific separations. The statistical approaches highlighted differences mainly in the presence and abundance of oxidoreductases, transferases, hydrolases, lyases, isomerases, and ligases. These differences are in line with the type of study conducted, based on the comparison of habitats with different environmental conditions. Within KO, MnoP was present with higher abundances in gorge samples. The MnoP is an outer membrane protein expressed only when there are low manganese levels. The *mnoP* gene is coordinated with the inner membrane transporter gene *mntH*. The Mur-binding site in the promoters of the *mntH* and *mnoP* genes allows occupancy in the presence of manganese and depletion when the metal is lacking^40^. Thanks to MnoP, Mn^2+^ can be translocated into proteoliposomes. Thus, it is essential for Mn^2+^ transport into cells and boosts the metals’ apparent affinity for cells^40^. Beyond these distinctions, PWY differences were mostly not significant. Differences were showed in PWY related to carbon metabolism and anthropogenic contaminants. The contribution of Mn to these PWY can be associated to the process of metals release induced by anthropogenic pollutants presence (e.g., organic contaminants) from aquifer sediments into groundwater^41^.

The more in-depth study of the predicted EC linked to Mn metabolism showed intriguing similarities. One of the most prevalent EC predicted with similar abundances in all the samples was cytochrome-c-oxidase, linked to Mn(II) oxidation^14^. Among the others, superoxide dismutase and peroxiredoxin were also shared by all samples. Superoxide dismutase and peroxiredoxins are linked to the oxidative stress response, mitigating superoxide and hydrogen peroxide excess, respectively, formed by Mn(IV) oxides bioreactions^42^. Moreover, superoxide dismutase isoenzymes can be overexpressed in different Mn reactions. Ercole et al., for example, reported that two superoxide dismutase isoenzymes are over expressed by *Arthrobacter* when cells are actively reducing MnO_2_ under aerobic conditions. Nevertheless, the same proteins are also expressed when reducing MnO_4_^-^ anaerobically^43^. Cd^2+^ exporting ATPase and sirohydrochlorin ferrochelatase, only present in “Grotta Grande dei Cervi” samples, are instead involved in metal stress tolerance^44^ and transport and cobalamin biosynthesis^45^. The differences among these enzymes highlighted different capabilities of the communities of the samples to be involved in carbon, sulphur, and nitrogen cycling and metals resistance and transport, without a clear separation between Gorge and Cave samples. These differences are strictly related to the adaptable and diverse nature of the microbes under variable temperature, nutrition availability, and pH within diverse ecological microbial niches, mediated by a spectrum of processes (e.g., DNA damage repair, recombination, conjugation, transformation, and transduction)^46^.

The PICRUSt 2 software is only a predictive tool of metagenome functions, and some rare environment-specific functions may not be identified. In our case, Mn metabolism functions were identified in line with other studies that described other deposits from extreme environments. Gonzalez-Pimentel et al., for example, compared four lava tubes of caves, identifying biogeochemical methane cycle gene copies^16^. Moreover, Chung et al. compared eleven sediment samples from the tailings drill cores in a tungsten mine, identifying cellular, environmental, and cellular processes among the others^47^. Further studies should be carried out to characterize the lithological and geological characteristics of the samples. Datation analyses and mineral characterizations would clarify the microbiological role in the deposition of these deposits and their evolutionary aspects. Comparing other black crusts belonging to different habitats and integrating the analysis with environmental conditions and geological surveys data will help to understand the common and specific Mn deposit formation based on ecosystem type. Only a multidisciplinary approach could unveil some aspects that contributed to the formation of these deposits. All the findings obtained with 16 s RNA gene metabarcoding (and bioinformatic tools) are associated with the moment of sampling and not to the evolution. Few attempts to sequence ancient transcriptomes have been done. However, future studies may be also directed toward RNA-seq analysis to evaluate the actual differentially expressed genes within the active community and validate the obtained findings.

## Methods

### Sampling

Samplings were carried out in two different major areas, the “Infernaccio” gorge (Viterbo, Italy) and the two cave systems (“Grotta Grande dei Cervi”—inactive speleogenesis—and “Ovito di Pietrasecca”—active speleogenesis) of the central portion of the Monti Carseolani ridge (Central Apennines, Italy). The complete description and photos of the sampling sites are provided in Supplementary information S1. Along the walls of gorge, four different samples were taken (G1-G4), collecting many replicates possible in the same sterile container. At sampling time, the temperature of the “Infernaccio” gorge (Supplementary Fig. S5) was 18 °C with a relative humidity of 60% (samples pH = 6.7). Same procedure was carried out for samples of “Grotta Grande dei Cervi” (sample C1 and C2) and “Ovito di Pietrasecca” (sample C3 and C4) caves (Supplementary Fig. S6), physicochemical parameters of the sampling sites were respectively 8 °C and 98% of relative humidity (sample pH of 7.1). Except for samples G1 and G3, black corroded sediments, all samples had solid structures (details summarized in Supplementary Table S10). All the samples were collected using a geologist hammer for rock samples from the gorge and sterile tools. All specimens were transported into a portable refrigerator from the sampling areas to the laboratory. Aliquots for molecular analyses were processed before storage. For each sample, five aliquots were selected, pooled together, and transferred into a solution of RNAlater (Ambion, Austin, TX, USA) according to the manufacturer's instructions. Aliquots for microscopic observations were transferred in sterile bags without treatments. All the samples were stored at − 80 °C until analysed.

### Sample characterization by SEM–EDS

Scanning electron microscopy (SEM) and energy-dispersive X-ray spectroscopy (EDS) were used to examine black deposits' morphological and geochemical properties from hypogean and epigean samples. Samples were examined by a Gemini500 scanning electron microscope (SEM) (Zeiss, Germany), and EDS microanalysis was carried out by an INCA X-ACT PELTIER COOLED detector (Aztec Energy, Oxford). SEM–EDS acquisitions were performed at different values of Electron High Tension (EHT), and Work Distance (WD) based on the sample observed (details are shown in the figures’ captions).

### DNA extraction and 16S rRNA analysis

Genomic DNA extraction was carried out, taking 500 mg of homogeneous samples, and extracting DNA with bead beating techniques according to the manufacturer’s protocol of the kit NucleoSpin®Soil (Macherey Nagel, Germany). Extracted samples were spectrophotometric and fluorometrically checked to evaluate DNA content and purity using a Nanodrop spectrophotometer (Thermo ScientificTM) and a Qubit fluorometer (Thermo ScientificTM). The different replicates were pooled together in an equimolar mixture for each sample. A specific 16S rRNA protocol was followed to amplify bacteria and archaea, using paired-end 16S rRNA community sequencing on the MiSeq Illumina platform (Bio-Fab Research, Italy). We focused on the V3 and V4 regions of 16S rRNA using the methodological approach previously described^12^. The reads were initially checked for quality and counted after filtering. QIIME2 (qiime2-2020.2 version; https://qiime2.org/)^13^ was used for ASV (Amplicon Sequence Variant) assembly with the DADA2 plugin version 1.18 (https://benjjneb.github.io/dada2/). From the 16S file obtained from the SILVA 132 database (https://www.arb-silva.de/ accessed on October 2021), the V3-V4 specific region was extracted and used for classifier training by the fit-classifier-naive-bayes plugin. Species accumulation curve and number of non-chimeric ASVs are presented in Supplementary Fig. S7 and Supplementary Table S9.

### Metagenome functions prediction

Based on 16S rRNA gene sequencing data, functional abundances of microbial communities were predicted by PICRUSt2 software (version 2.0; https://huttenhower.sph.harvard.edu/picrust/)^11^. PICRUSt2 predictions were based on ASVs sequence profiles/abundances (BIOM file format obtained from qiime2 with a frequency filter of 100). Three gene family databases were used, Kyoto Encyclopedia of Genes and Genomes (KEGG), orthologs (KO), Enzyme Commission numbers (EC) and MetaCyc pathways abundances^48–50^. PICRUSt2 outputs (KO, EC, PWY) were analysed by QIIME2 software. The presence of enzymes involved in redox biotransformation, metal resistance, and respiratory processes was also verified in the EC outputs to learn more about these activities following (Supplementary Table S11)^14^.

### Statistical analysis

Primer statistical software (version 7; https://www.primer-e.com/our-software/primer-version-7/) was used to calculate alpha-diversity metrics (i.e., Simpson, Shannon and Chao1 indices) and build taxonomy barplots of ASVs at Phylum, Class, and Genus levels. To underline the common ASVs within the different samples, Venn diagrams were built by Bioinformatics & Evolutionary Genomics tool (https://bioinformatics.psb.ugent.be/webtools/Venn/). PICRUSt outputs were analysed by the ALDEx2 package (version 1.29.2; https://bioconductor.unipi.it/packages/3.15/bioc/html/ALDEx2.html)^51–53^ to perform comparison tests between gorge and cave samples (generalized linear model—glm) and to plot the results into effect (MW) plot for each PICRUSt2 output. We underlined the most representative metabolic pathways and their correlation between the different samples, generating a heatmap elaborated with Primer 7 software.

## Supplementary Information

Supplementary Information.

## Data Availability

The datasets generated during and/or analysed during the current study are available from the corresponding author on reasonable request. The nucleotide sequences of the partial 16S rRNA gene segments determined in this study have been deposited in the Sequence Read Archive of NCBI database repository, BioProject: PRJNA838991 (http://www.ncbi.nlm.nih.gov/bioproject/838991).
