# Supplementary material for: Exploring structure, microbiota, and metagenome functions of epigean and hypogean black deposits by microscopic, molecular and bioinformatic approaches
Source: Sci Rep. 2022 Nov 12;12:19405. doi: 10.1038/s41598-022-24159-9 (PMC9653421; doi:10.1038/s41598-022-24159-9)
Supplement: Supplementary file 1 — Supplementary Information. [file 41598_2022_24159_MOESM1_ESM.docx]

**Supplementary information**

**Exploring structure, microbiota, and metagenome functions of epigean and hypogean black deposits by microscopic, molecular and bioinformatic approaches**

**Beatrice Farda^1^, Ilaria Vaccarelli^1^, Claudia Ercole^1^, Rihab Djebaili^1^, Maddalena Del Gallo^1^, Marika Pellegrini^1,*^**

^1^Department of Life, Health and Environmental Sciences, University of L'Aquila, L'Aquila, Italy

* Correspondence:
marika.pellegrini@univaq.it

**Supplementary Figure 1** SEM-EDS maps and details of the elements’ distribution for gorge samples.

**G1 G2**


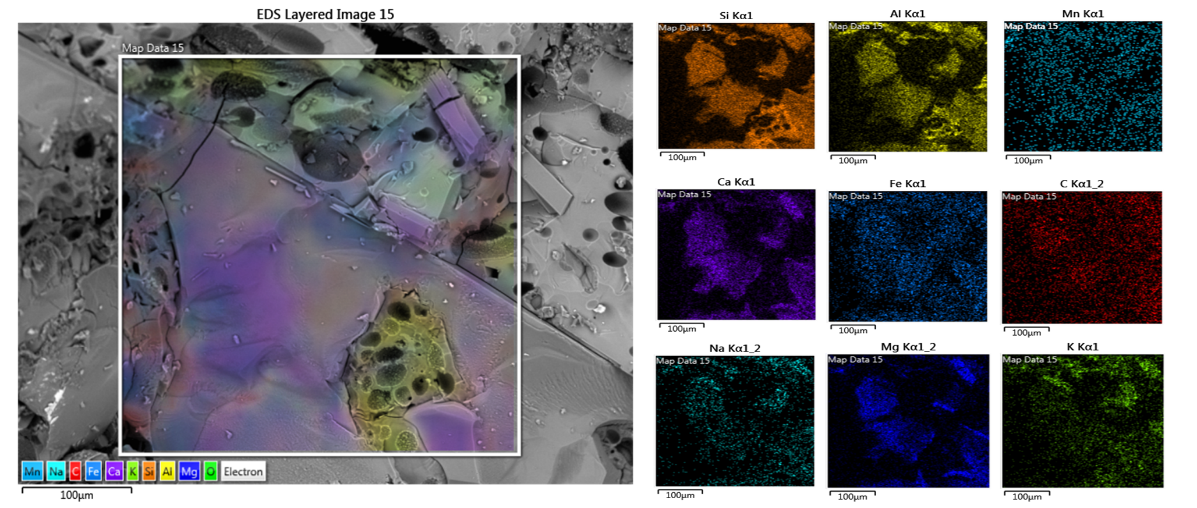

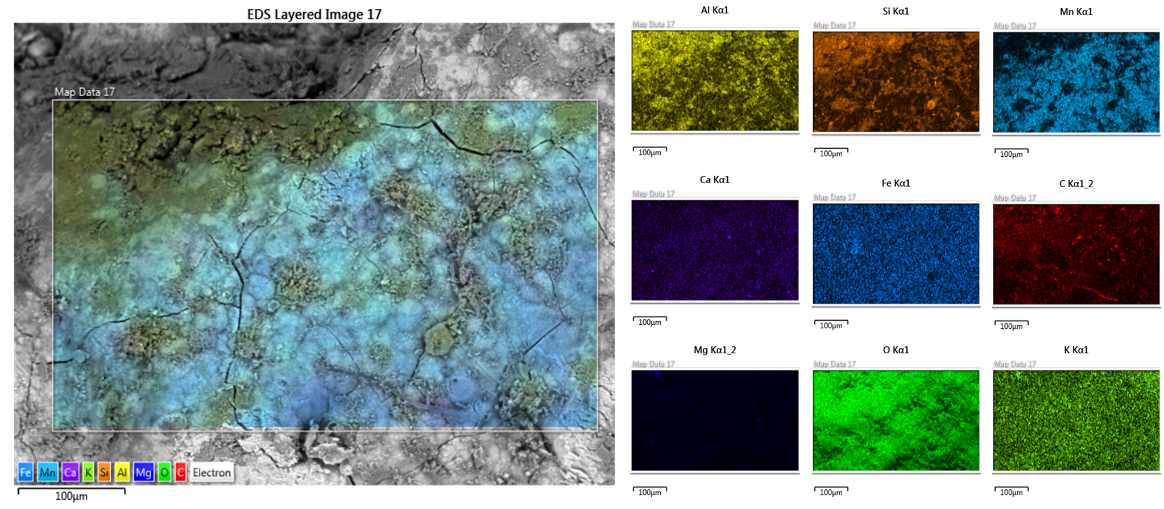


**G3 G4**


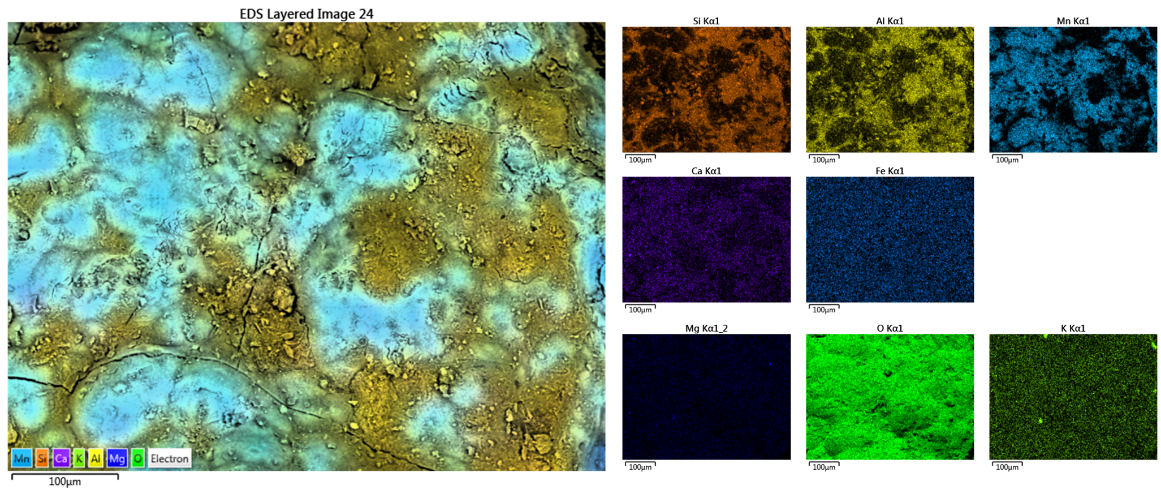

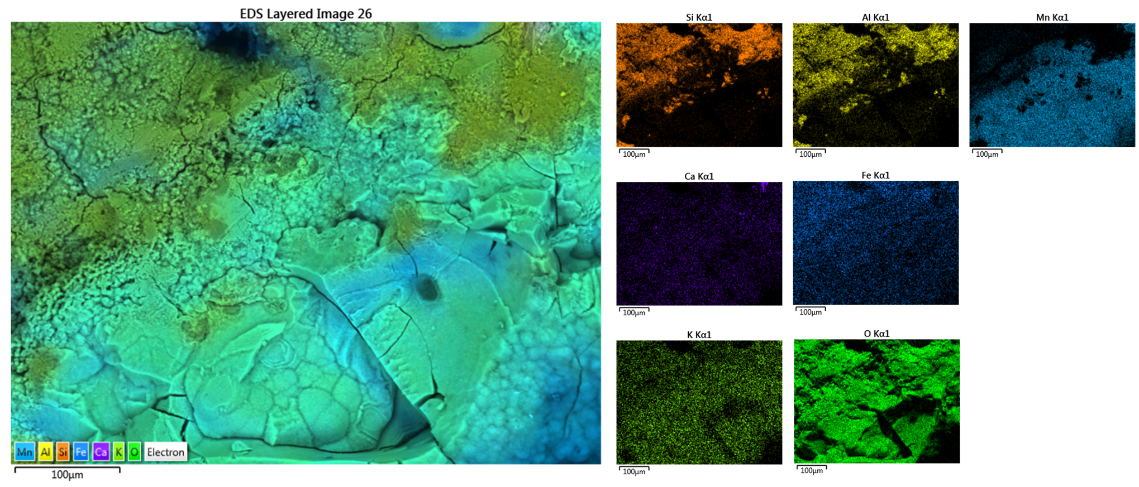


**Supplementary Figure 2** SEM-EDS maps and details of the elements’ distribution for cave samples.

**C1 C2**


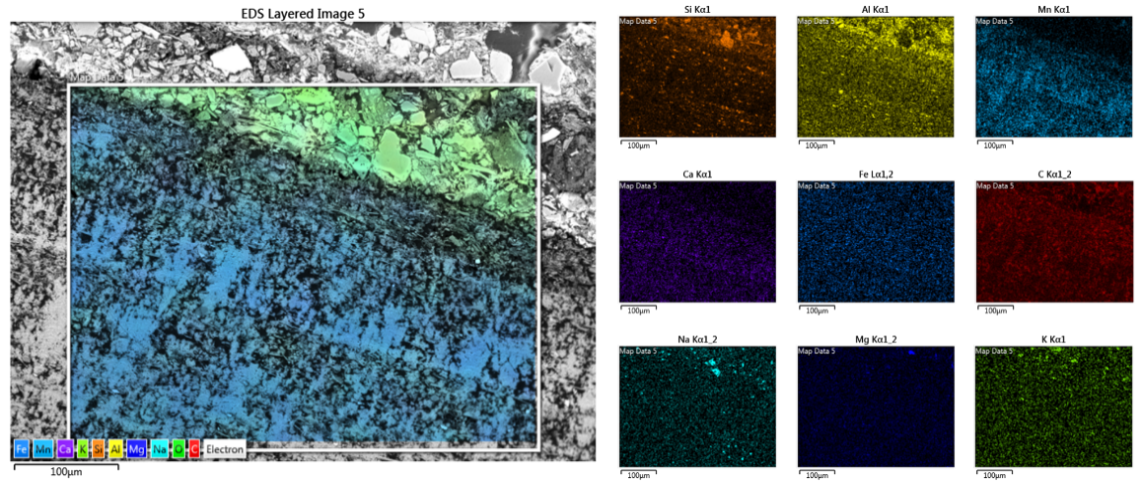
 **
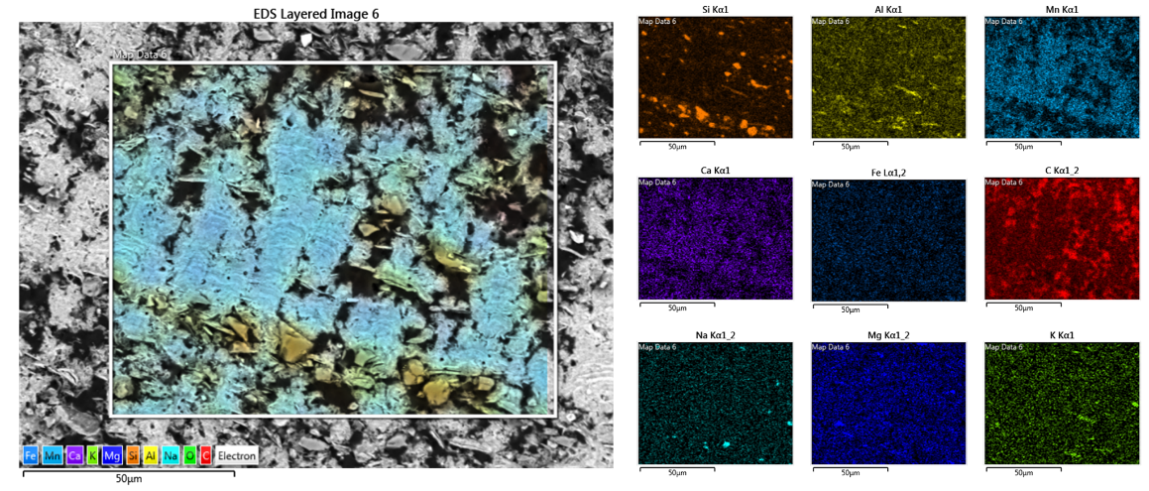
**

**C3 C4**


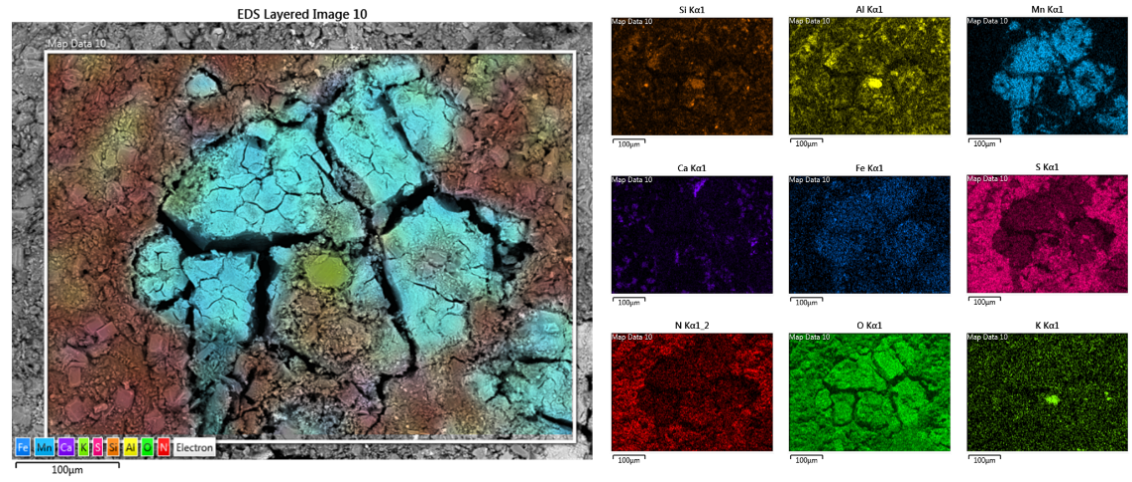

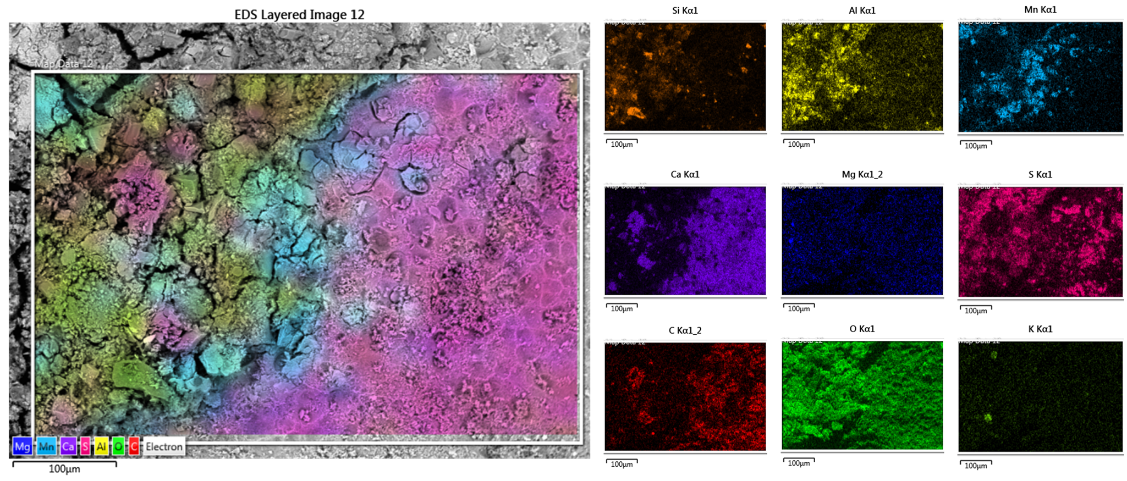


**Supplementary Figure 3** Dendrograms of cluster analyses showing the similarities among the samples based on enzymes (A), keg orthology (B), and pathways (C) predicted by PICURUSt software.


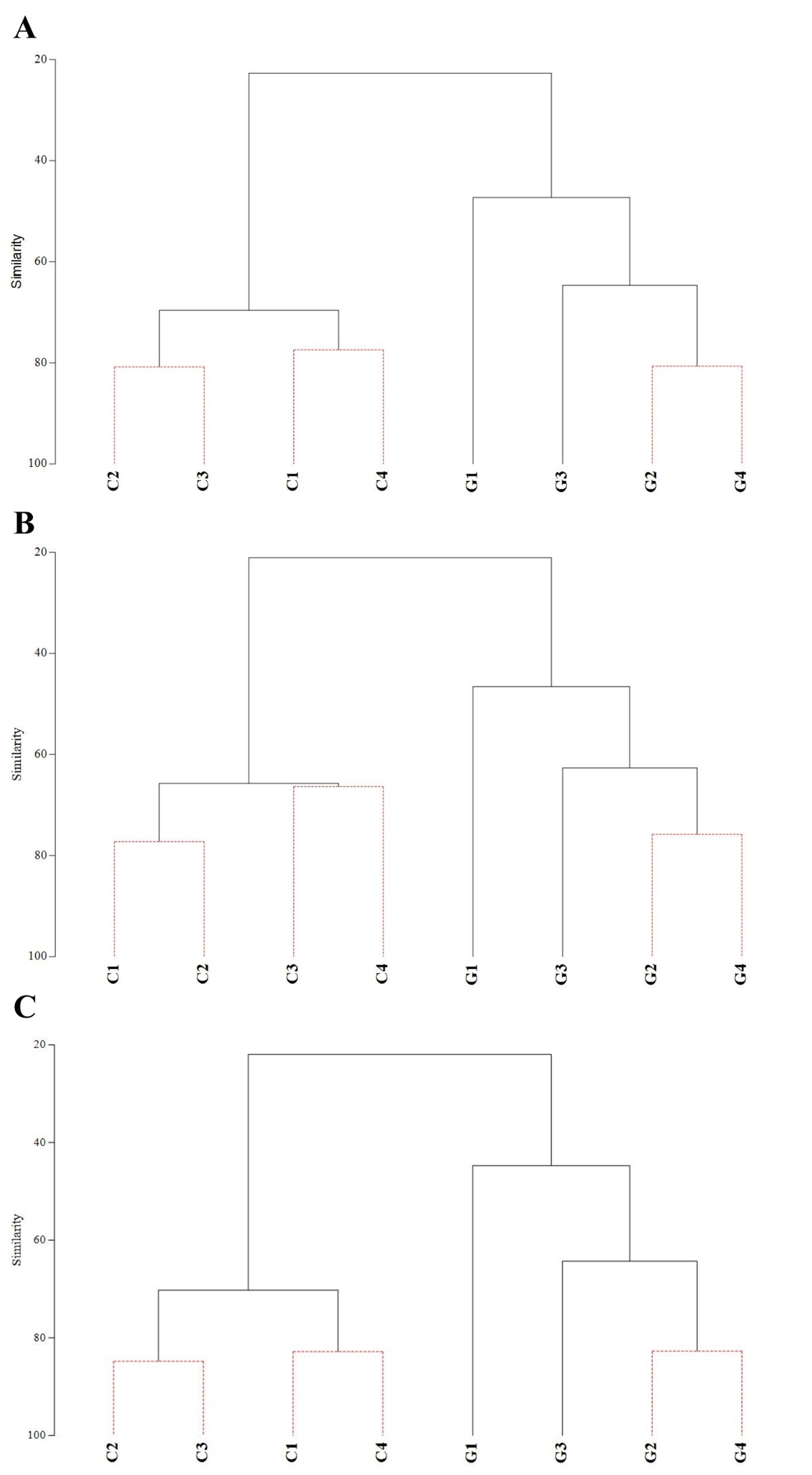


**Supplementary Figure 4** Principal component analysis 3D plot of observations on the three axis PC1, PC2 and PC3.


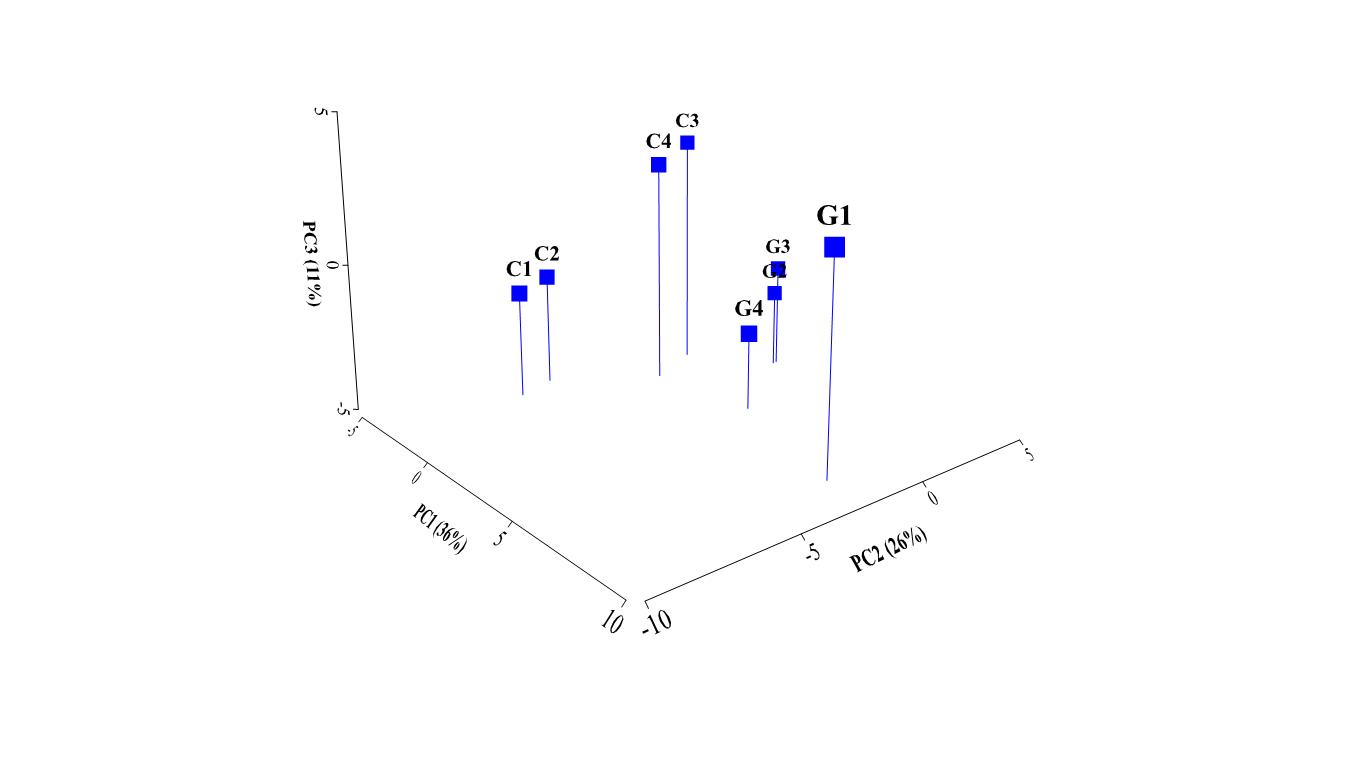


**
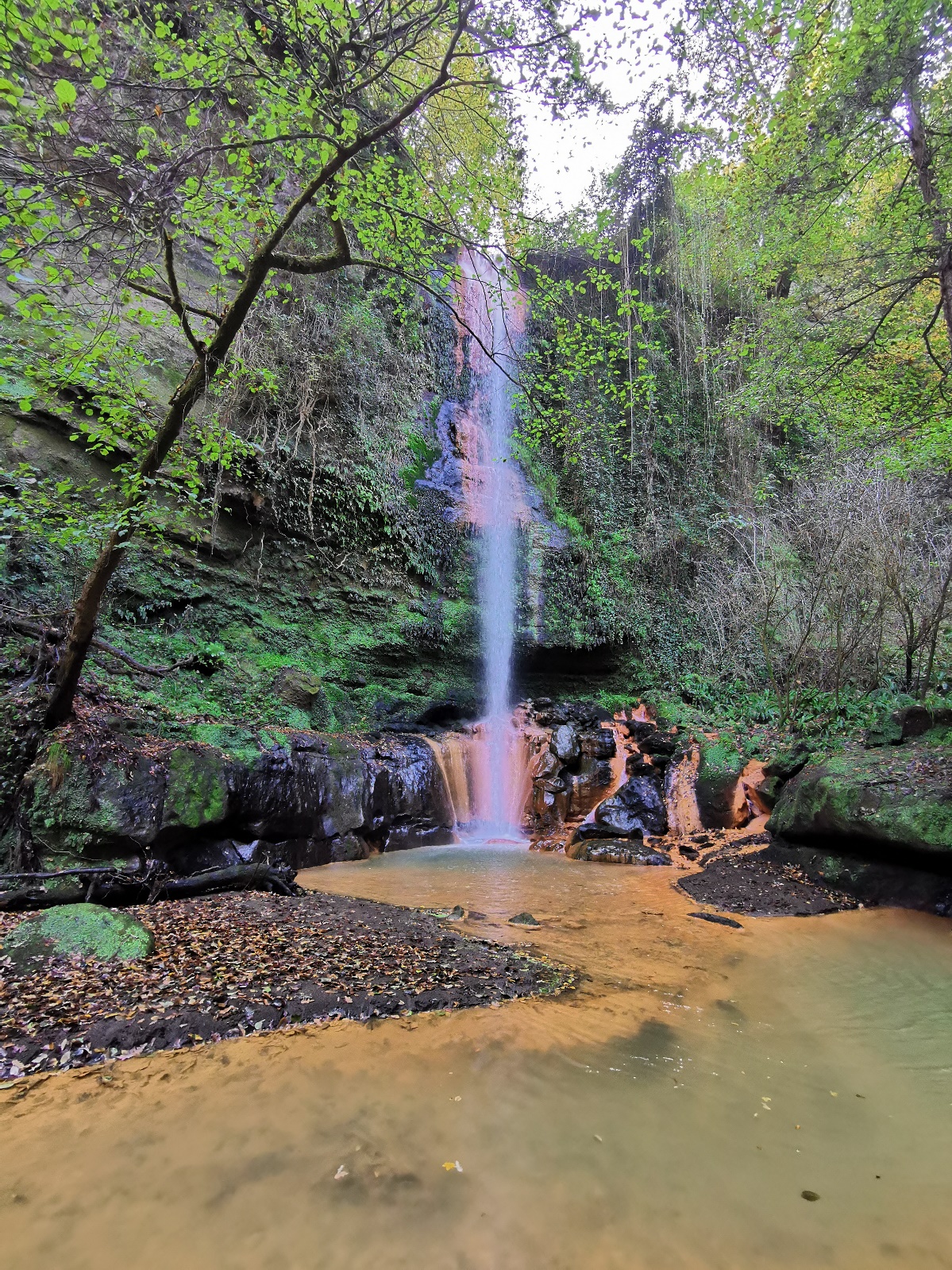
 Supplementary Figure 5** Sites of “Infernaccio” gorge (Viterbo, Italy) were samples G1-G4 were collected. Photo by Simone Chiani. The gorge’s samples sampling site near Infernaccios’s falls, located in the Viterbo province (Lazio, Italy - 42°31'36.57"N; 12° 7'40.70"E), is situated within the “Tuscia” area, which develops mostly on volcanic substrates deriving from the explosive activity of three important volcanic complexes (Vulsino, Vicano, and Cimino). The low and monotonous tufaceous plateaus develop between these main apparatuses, which are modest in height, furrowed by deep valleys called Gorges. The volcanic soils develop on older soils of sedimentary origin, which can outcrop or emerge from the volcanic cover in a rather small way. In fact, in ancient times, less than 2 million years ago, the waters of the Pliocene Sea covered all this newly emerged area, lapping the Apennine chain. The territory was modified during the Pleistocene, in which the simultaneous marine regression and genesis of the three volcanic complexes led to the territory being covered by lava and ignimbrite deposits, subsequently subject to degradation. Currently, three types of soils can be distinguished in the territory: soils of magmatic pyroclastic origin, mainly coherent products consisting of volcanic and sedimentary rocks of variable size and limited extension in outcrop; pyroclastic flows with a cineritic-pomiceous matrix and launch pyroclastics consisting of lapillaceous-sandy and cineritic levels. The erosive action on the young watercourses' soft and friable volcanic tuff substrate has given rise to the deep incisions called "ravines", that is, gullies dug in the pyroclastic substrata by the erosion of water in regimes of strong flow, as in the post-glacial period. The most recent manifestation of the phenomenon is evident in the very high slopes. Due to an extensive and branched hydrographic network and low resistance to erosive agents of pyroclastic products, gorges are a peculiar and defining component of the Viterbo province landscape.


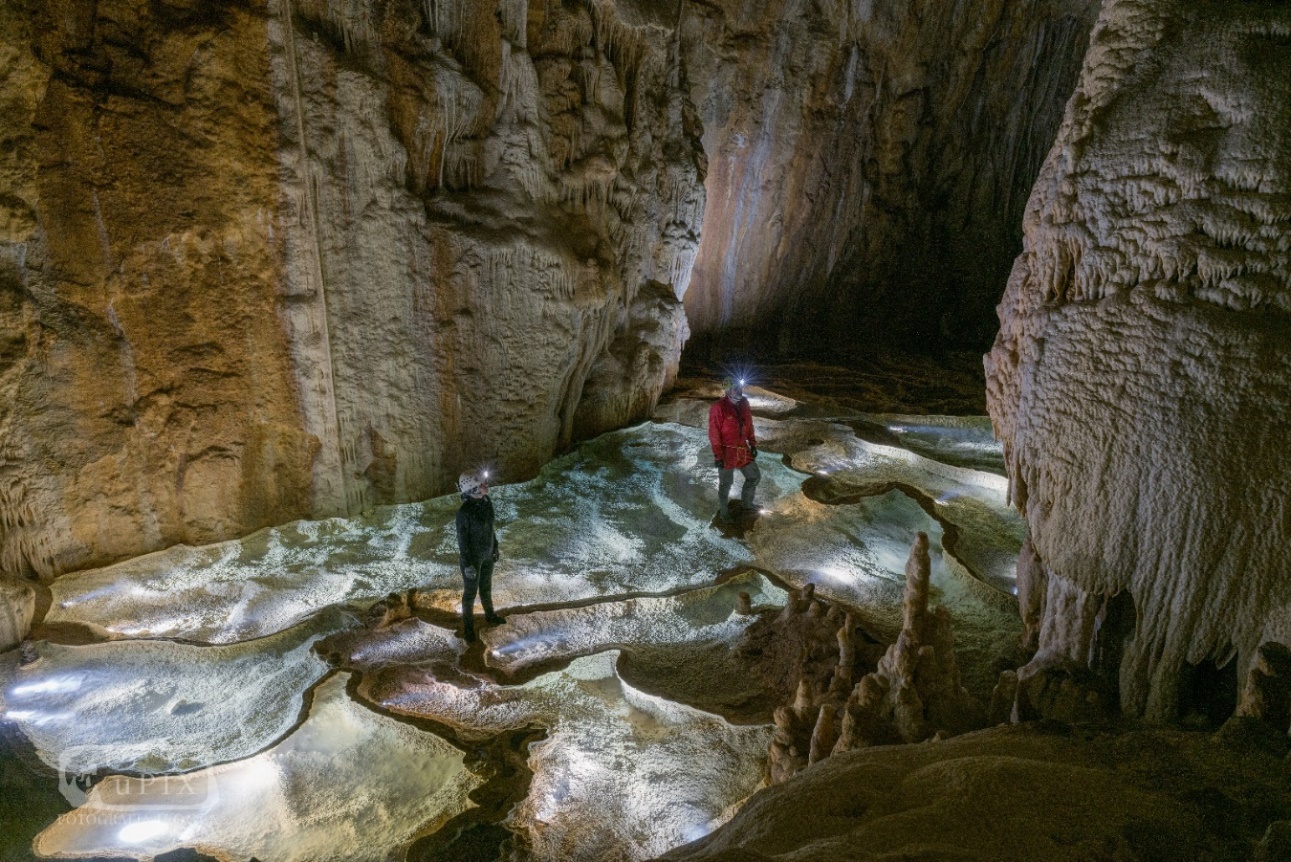

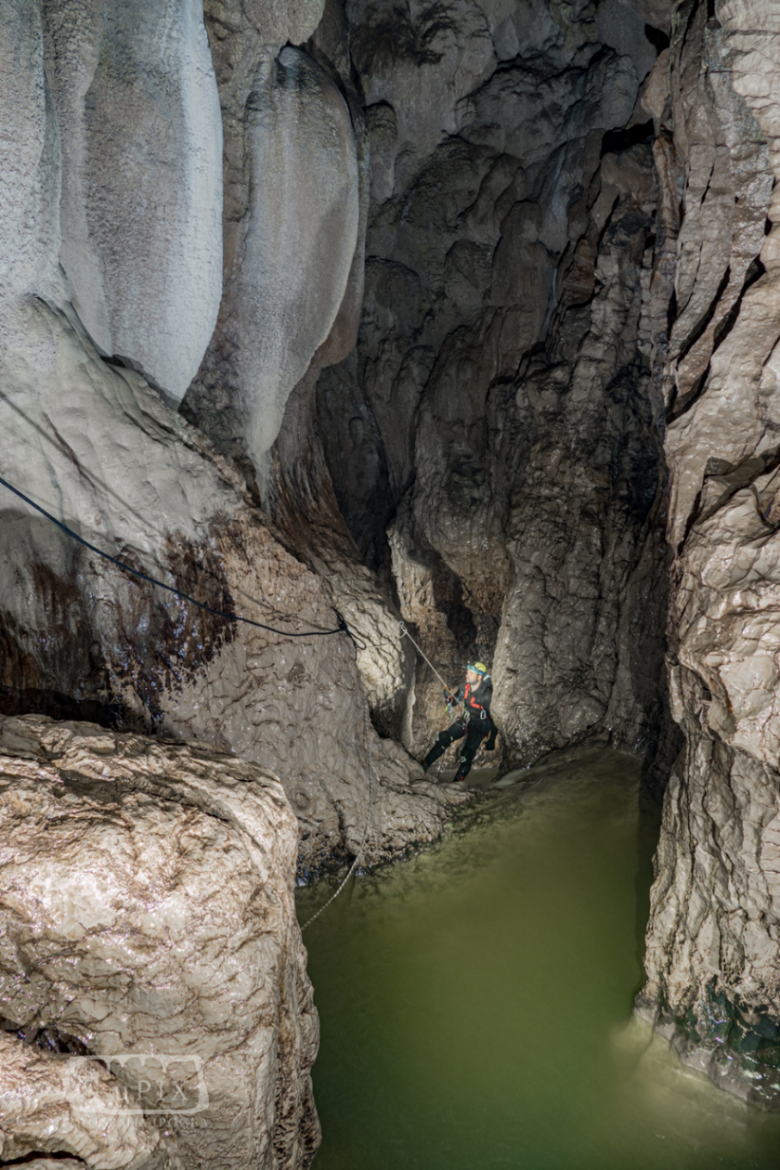


**Supplementary Figure 6** Sites of “Grotta grande dei Cervi” (on the left) and of “Pietrasecca” (on the right) where samples C1-2 and C3-C4 were collected, respectively. Photo by uPIX—uNDER PIXel Fotografia Ipogea. Cave samples were taken in the central portion of the Monti Carseolani ridge (Central Apennines). This chain is located on the border between Lazio and Abruzzo and runs from NW to SE for about 25 km, between Mount Velino to the NE and the Simbruini Mountains to the SW. In the carbonate ridge of Pietrasecca, falling in this area, a well-structured system of caves develops, known as the karst system of Pietrasecca, of which the “Grotta Grande dei Cervi” (samples C1 and C2) and the “Ovito di Pietrasecca” (samples C3 and C4) represent the most important cavities. The latter is closely connected from an evolutionary and hydrogeological point of view. The entire system evolved in a ridge formed by limestone dating back to the Cretaceous-Miocene and more recent flysch deposits, delimited by faults with a NE-SE trend. The cavities under examination proceed along with these fractures, which develop transversally to the ridge. “Grotta Grande dei Cervi” entrance opens at an altitude of 858 m above sea level (42° 08' 09'' N - 13° 07' 43'' E). The entrance has a height difference of 107 m and a horizontal development of 1875 m. This cavity represents the most ancient point of capture of the waters of the karstic system, now active with the “Ovito di Pietrasecca”. The latter is located at 908 m above sea level. (42° 08′ 0'' N - 13° 07′ 45'' E), has a total height difference of about 70 m and total development of about 1200 m. The initial portion of the Ovito di Pietrasecca appears as a Gorge, revealing a rapid development under a hydraulic gradient not very different from the present one. In general, both systems have much erosive activity, which is driven by considerable water and solid transport.

**Supplementary Figure 7** Alpha diversity rarefaction curves of the microbial richness in the different samples according to their respective sample size (PAST 4.03).


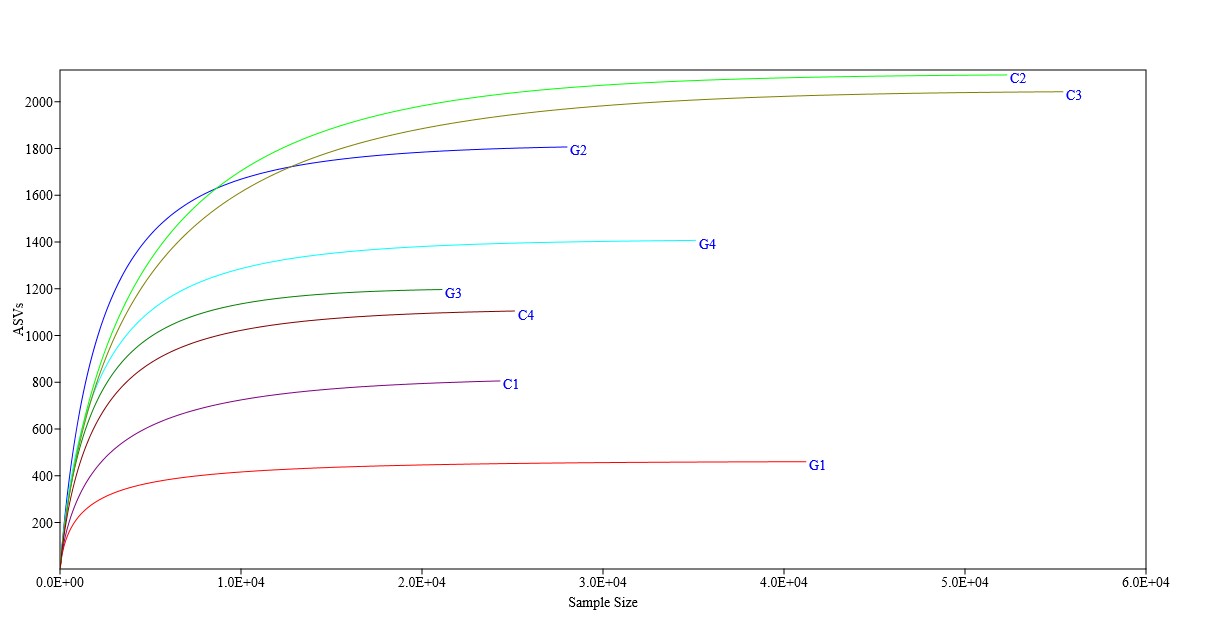


**Supplementary Table 1** ASVs abundances (%) found at the domain level.

| **G1** | **G2** | **G3** | **G4** | **C1** | **C2** | **C3** | **C4** | **Domain** |
| --- | --- | --- | --- | --- | --- | --- | --- | --- |
| 26.5 | 0.3 | 0.1 | 0.4 | 0.0 | 1.0 | 2.5 | 3.3 | Archaea |
| 73.5 | 99.7 | 99.9 | 99.6 | 100.0 | 99.0 | 97.5 | 96.7 | Bacteria |

**Supplementary Table 2** Total number of different ASVs belonging to each phylum.

| **Phylum** | **G1** | **G2** | **G3** | **G4** | **C1** | **C2** | **C3** | **C4** |
| --- | --- | --- | --- | --- | --- | --- | --- | --- |
| Acidobacteriota | 46 | 256 | 93 | 144 | 87 | 325 | 276 | 147 |
| Actinobacteriota | 73 | 79 | 50 | 356 | 78 | 106 | 55 |  |
| Bacteroidota |  | 202 | 172 |  | 39 |  | 120 |  |
| Chloroflexota | 36 | 101 | 60 | 100 | 73 | 224 | 135 | 90 |
| Crenarchaeota | 19 |  |  |  |  |  |  |  |
| Bacillota |  |  |  |  | 82 | 153 |  | 45 |
| Gemmatimonadota |  | 90 | 28 |  |  | 59 |  |  |
| Methylomirabilota |  |  |  |  |  | 60 | 48 |  |
| Micrarchaeota | 20 |  |  |  |  |  |  |  |
| Myxococcota |  | 85 | 64 |  |  |  |  |  |
| Nitrospirota |  | 36 | 32 |  |  |  | 26 | 23 |
| Patescibacteria | 52 |  | 94 | 168 |  |  | 166 |  |
| Planctomycetota |  | 124 |  |  | 59 | 201 | 235 | 122 |
| Pseudomonadota | 79 | 435 | 329 | 219 | 223 | 287 | 339 | 244 |
| Thermoplasmatota | 8 |  |  |  |  |  |  |  |
| Verrucomicrobiota |  | 98 | 63 | 87 |  |  |  |  |

**Supplementary Table 3** Venn Diagram details for the common ASVs at the phylum level.

| **Group** | **N** | **Taxa** |
| --- | --- | --- |
| **GORGE** | | |
| G1 G2 G3 G4 | 4 | Actinobacteriota PseudomonadotaChloroflexotaAcidobacteriota |
| G2 G3 G4 | 1 | Verrucomicrobiota |
| G1 G4 | 1 | Patescibacteria |
| G2 G3 | 5 | Nitrospirota Myxococcota Planctomycetota Bacteroidota Gemmatimonadota |
| G1 | 3 | Micrarchaeota Thermoplasmatota Crenarchaeota |
| **CAVE** | | |
| C1 C2 C3 C4 | 4 | PseudomonadotaChloroflexotaAcidobacteriota Planctomycetota |
| C1 C2 C3 | 1 | Actinobacteriota |
| C1 C2 C4 | 1 | Firmicutes |
| C1 C3 | 1 | Bacteroidota |
| C2 C3 | 1 | Methylomirabilota |
| C3 C4 | 1 | Nitrospirota |
| C2 | 1 | Gemmatimonadota |
| C3 | 1 | Patescibacteria |
| **GORGE vs CAVE** | | |
| CAVE GORGE | 9 | Nitrospirota Actinobacteriota PseudomonadotaChloroflexotaPatescibacteria Acidobacteriota Planctomycetota Bacteroidota Gemmatimonadota |
| GORGE | 5 | Micrarchaeota Verrucomicrobiota Thermoplasmatota Myxococcota Crenarchaeota |
| CAVE | 2 | BacillotaMethylomirabilota |

**Supplementary Table 4** Venn Diagram details for the common ASVs at the class level.

| **Group** | **N** | **Taxa** |
| --- | --- | --- |
| **GORGE** | | |
| G1 G2 G3 G4 | 2 | AlphaPseudomonadota GammaPseudomonadota |
| G2 G3 G4 | 1 | Verrucomicrobiae |
| G1 G2 | 1 | Acidobacteriae |
| G1 G3 | 1 | Parcubacteria |
| G1 G4 | 3 | Acidimicrobiia Actinobacteria Saccharimonadia |
| G2 G3 | 2 | Bacteroidia Nitrospiria |
| G1 | 4 | AD3 Nitrososphaeria Thermoplasmata Micrarchaeia |
| G2 | 3 | Vicinamibacteria Gemmatimonadetes Polyangia |
| G4 | 3 | Thermoleophilia MB-A2-108 Blastocatellia |
| **CAVE** | | |
| C1 C2 C3 C4 | 2 | AlphaPseudomonadota GammaPseudomonadota |
| C1 C2 C4 | 1 | Bacilli |
| C1 C3 | 1 | Bacteroidia |
| C2 C3 | 2 | Acidobacteriae Methylomirabilia |
| C3 C4 | 1 | Nitrospiria |
| C1 | 1 | Actinobacteria |
| C2 | 2 | Vicinamibacteria Gemmatimonadetes |
| C3 | 1 | Blastocatellia |
| C4 | 1 | Anaerolineae |
| **GORGE vs CAVE** | | |
| CAVE GORGE | 9 | Vicinamibacteria AlphaPseudomonadota Bacteroidia Gemmatimonadetes Actinobacteria Acidobacteriae GammaPseudomonadota Blastocatellia Nitrospiria |
| GORGE | 11 | Verrucomicrobiae AD3 Thermoleophilia Nitrososphaeria MB-A2-108 Acidimicrobiia Thermoplasmata Saccharimonadia Parcubacteria Micrarchaeia Polyangia |
| CAVE | 3 | Methylomirabilia Anaerolineae Bacilli |

**Supplementary Table 5** Venn Diagram details for the common ASVs at the genus level.

| **Group** | **N** | **Taxa** |
| --- | --- | --- |
| **GORGE** | | |
| G1 G2 G3 G4 | 1 | Uncultured |
| G2 G3 G4 | 1 | Unknown |
| G2 G3 | 1 | *Nitrospira* |
| G1 | 8 | *Subgroup_13 AD3 Nitrosotaleaceae Candidatus_Micrarchaeum Acidothermus KF-JG30-C25 Group_1.1c LWQ8* |
| G2 | 3 | *TRA3-20 Vicinamibacteraceae MND1* |
| G3 | 1 | *Crenothrix* |
| G4 | 4 | *IMCC26256 RB41 Candidatus_Udaeobacter MB-A2-108* |
| **CAVE** | | |
| C1 C2 C3 C4 | 1 | Unknown |
| C1 C2 C4 | 1 | *Bacillus* |
| C2 C3 C4 | 2 | *wb1-P19* Uncultured |
| C3 C4 | 1 | *Nitrospira* |
| C1 | 3 | *Massilia Flavobacterium Pseudomonas* |
| C3 | 1 | *PLTA13* |
| **GORGE vs CAVE** | | |
| CAVE GORGE | 3 | Uncultured *Nitrospira* Unknown |
| GORGE | 16 | *TRA3-20 Subgroup_13 IMCC26256 RB41 AD3 Nitrosotaleaceae Candidatus_Udaeobacter MB-A2-108 Candidatus_Micrarchaeum Vicinamibacteraceae Acidothermus MND1 KF-JG30-C25 Crenothrix Group_1.1c LWQ8* |
| CAVE | 6 | *PLTA13 Flavobacterium Massilia Bacillus wb1-P19 Pseudomonas* |

**Supplementary Table 6** Export of differences obtained from the statistical comparison of gorge and cave samples EC abundances.

| **glm.ep** | **glm.eBH** | **rab.all** | **rab.win.Forra** | **rab.win.Grotta** | **diff.btw** | **EC** | **Name** | **Class** |
| --- | --- | --- | --- | --- | --- | --- | --- | --- |
| 0.002 | 0.042 | -3.209 | -0.354 | -8.285 | -7.897 | EC:1.1.1.333 | Decaprenylphospho-beta-D-erythro-pentofuranosid-2-ulose 2-reductase. | Oxidoreductases |
| 0.002 | 0.034 | -6.525 | -2.409 | -9.806 | -7.636 | EC:1.1.1.56 | ribitol 2-dehydrogenase | Oxidoreductases |
| 0.000 | 0.021 | -4.007 | -0.220 | -10.291 | -10.571 | EC:1.1.1.65 | pyridoxine 4-dehydrogenase | Oxidoreductases |
| 0.002 | 0.040 | -3.237 | -0.324 | -8.333 | -7.924 | EC:1.1.98.3 | decaprenylphospho-beta-D-ribofuranose 2-dehydrogenase | Oxidoreductases |
| 0.001 | 0.033 | -4.850 | -1.579 | -10.228 | -9.732 | EC:1.14.13.114 | 6-hydroxynicotinate 3-monooxygenase | Oxidoreductases |
| 0.002 | 0.041 | -2.503 | -6.941 | 0.074 | 6.985 | EC:1.8.98.1 | dihydromethanophenazine:CoB-CoM heterodisulfide reductase | Oxidoreductases |
| 0.000 | 0.018 | -0.248 | -2.668 | 0.751 | 3.415 | EC:2.1.1.173 | 23S rRNA (guanine2445-N2)-methyltransferase | Transferases |
| 0.000 | 0.018 | -0.007 | -2.593 | 2.538 | 5.294 | EC:2.1.1.200 | tRNA (cytidine32/uridine32-2'-O)-methyltransferase | Transferases |
| 0.000 | 0.015 | 0.029 | -2.529 | 2.424 | 4.996 | EC:2.1.1.242 | 16S rRNA (guanine1516-N2)-methyltransferase | Transferases |
| 0.001 | 0.021 | -0.270 | -2.630 | 0.758 | 3.359 | EC:2.1.1.264 | 23S rRNA (guanine2069-N7)-methyltransferase | Transferases |
| 0.001 | 0.028 | 2.293 | -0.260 | 3.656 | 4.045 | EC:2.3.1.46 | homoserine O-succinyltransferase | Transferases |
| 0.001 | 0.035 | 1.350 | 0.437 | 3.038 | 2.496 | EC:2.3.2.3 | lysyltransferase | Transferases |
| 0.001 | 0.041 | 3.607 | 2.871 | 4.304 | 1.407 | EC:2.4.2.1 | purine-nucleoside phosphorylase | Transferases |
| 0.002 | 0.035 | -0.478 | -3.365 | 2.508 | 6.170 | EC:2.4.2.44 | S-methyl-5'-thioinosine phosphorylase | Transferases |
| 0.002 | 0.040 | -3.265 | -0.317 | -8.318 | -7.842 | EC:2.4.2.45 | decaprenyl-phosphate phosphoribosyltransferase | Transferases |
| 0.000 | 0.020 | -4.063 | -0.369 | -10.367 | -10.829 | EC:2.7.1.119 | hygromycin-B 7''-O-kinase | Transferases |
| 0.000 | 0.013 | 4.359 | 3.517 | 4.680 | 1.271 | EC:2.7.1.4 | fructokinase | Transferases |
| 0.002 | 0.037 | -6.574 | -1.612 | -8.847 | -7.159 | EC:2.7.1.47 | D-ribulokinase | Transferases |
| 0.001 | 0.036 | -2.668 | -1.574 | -4.348 | -2.757 | EC:3.1.1.73 | feruloyl esterase | Hydrolases |
| 0.000 | 0.019 | -4.216 | -0.041 | -10.370 | -11.124 | EC:3.2.1.58 | glucan 1,3-beta-glucosidase | Hydrolases |
| 0.000 | 0.018 | 1.930 | 0.964 | 3.088 | 2.165 | EC:3.4.13.9 | Xaa-Pro dipeptidase | Hydrolases |
| 0.000 | 0.014 | -3.590 | 0.802 | -10.396 | -11.940 | EC:3.5.1.77 | N-carbamoyl-D-amino-acid hydrolase | Hydrolases |
| 0.000 | 0.021 | -3.750 | -0.345 | -10.547 | -10.998 | EC:3.5.1.82 | N-acyl-D-glutamate deacylase | Hydrolases |
| 0.000 | 0.022 | -3.461 | 0.101 | -10.158 | -11.212 | EC:3.5.2.12 | 6-aminohexanoate-cyclic-dimer hydrolase | Hydrolases |
| 0.000 | 0.019 | -3.995 | -0.330 | -10.377 | -10.915 | EC:3.5.2.15 | cyanuric acid amidohydrolase | Hydrolases |
| 0.003 | 0.041 | -2.485 | -4.738 | -0.507 | 4.060 | EC:4.1.2.20 | 2-dehydro-3-deoxyglucarate aldolase | Lyases |
| 0.001 | 0.038 | 0.872 | 1.551 | -1.216 | -2.774 | EC:4.1.3.24 | malyl-CoA lyase | Lyases |
| 0.002 | 0.039 | -2.759 | -8.559 | -1.181 | 8.214 | EC:4.2.1.32 | L(+)-tartrate dehydratase | Lyases |
| 0.001 | 0.036 | -3.160 | -0.222 | -9.127 | -9.106 | EC:4.4.1.22 | S-(hydroxymethyl)glutathione synthase | Lyases |
| 0.002 | 0.044 | -1.837 | -6.763 | 0.258 | 7.109 | EC:4.4.1.24 | (2R)-sulfolactate sulfo-lyase | Lyases |
| 0.001 | 0.031 | -4.892 | -1.487 | -10.354 | -10.014 | EC:4.6.1.2 | guanylate cyclase | Lyases |
| 0.000 | 0.019 | -4.115 | -0.173 | -10.356 | -10.719 | EC:5.1.2.2 | mandelate racemase | Isomerases |
| 0.002 | 0.049 | 2.111 | 1.255 | 3.126 | 1.796 | EC:5.4.99.19 | 16S rRNA pseudouridine516 synthase | Isomerases |
| 0.003 | 0.049 | 0.122 | -3.332 | 2.594 | 5.862 | EC:5.4.99.27 | tRNA pseudouridine13 synthase | Isomerases |
| 0.000 | 0.022 | -4.106 | -0.262 | -10.418 | -10.887 | EC:6.2.1.27 | 4-hydroxybenzoate---CoA ligase | Ligases |
|  |  |  |  |  |  |  |  |  |

**Supplementary Table 7** Export of differences obtained from the statistical comparison of gorge and cave samples KO abundances.

| **glm.ep** | **glm.eBH** | **rab.all** | **rab.win.Forra** | **rab.win.Grotta** | **diff.btw** | **KO** | **Name** |
| --- | --- | --- | --- | --- | --- | --- | --- |
| 0.00 | 0.00 | 1.01 | 3.52 | -1.27 | -4.77 | K13572 | proteasome accessory factor B |
| 0.00 | 0.01 | -2.31 | 2.27 | -9.08 | -12.05 | K01459 | N-carbamoyl-D-amino-acid hydrolase |
| 0.00 | 0.02 | -2.66 | 1.85 | -9.35 | -11.91 | K16080 | **high affinity Mn2+ porin** |
| 0.00 | 0.02 | 2.18 | 4.72 | 0.67 | -3.90 | K18955 | WhiB family transcriptional regulator, redox-sensing transcriptional regulator |
| 0.00 | 0.02 | -3.08 | 1.26 | -9.46 | -11.58 | K01210 | glucan 1,3-beta-glucosidase |
| 0.00 | 0.02 | 3.70 | 4.47 | 3.05 | -1.32 | K01880 | glycyl-tRNA synthetase |
| 0.00 | 0.02 | -0.88 | 2.61 | -2.68 | -5.09 | K07653 | two-component system, OmpR family, sensor histidine kinase MprB |
| 0.00 | 0.02 | 3.53 | 4.23 | 3.05 | -1.14 | K00130 | betaine-aldehyde dehydrogenase |
| 0.00 | 0.02 | -3.01 | 1.12 | -9.36 | -11.50 | K05818 | IclR family transcriptional regulator, mhp operon transcriptional activator |
| 0.00 | 0.02 | 1.14 | -1.26 | 3.38 | 4.65 | K15984 | 16S rRNA (guanine1516-N2)-methyltransferase |
| 0.00 | 0.02 | -2.88 | 1.12 | -9.41 | -11.39 | K03383 | cyanuric acid amidohydrolase |
| 0.00 | 0.02 | -3.13 | 0.95 | -9.31 | -11.30 | K01781 | mandelate racemase |
| 0.00 | 0.02 | 2.06 | 3.62 | 1.13 | -2.43 | K07004 | uncharacterized protein |
| 0.00 | 0.02 | -3.14 | 0.99 | -9.36 | -11.27 | K09477 | citrate:succinate antiporter |
| 0.00 | 0.02 | -3.09 | 1.01 | -9.44 | -11.30 | K05275 | pyridoxine 4-dehydrogenase |
| 0.00 | 0.02 | -3.05 | 1.00 | -9.33 | -11.18 | K04105 | 4-hydroxybenzoate-CoA ligase |
| 0.00 | 0.02 | -3.06 | 1.04 | -9.36 | -11.30 | K06912 | alpha-ketoglutarate-dependent 2,4-dichlorophenoxyacetate dioxygenase |
| 0.00 | 0.02 | 0.92 | -1.41 | 3.37 | 4.79 | K07153 | high frequency lysogenization protein |
| 0.00 | 0.02 | -2.79 | 1.07 | -9.29 | -11.08 | K17880 | hygromycin-B 7''-O-kinase |
| 0.00 | 0.02 | 1.09 | -1.33 | 3.34 | 4.77 | K03528 | cell division protein ZipA |
| 0.00 | 0.02 | -2.96 | 1.00 | -9.32 | -11.13 | K11106 | L-tartrate/succinate antiporter |
| 0.00 | 0.02 | -3.24 | 1.12 | -9.38 | -10.96 | K15468 | cytochrome P450 family 107 subfamily K polypeptide 1 |
| 0.00 | 0.02 | -2.28 | 1.54 | -9.39 | -11.59 | K01471 | 6-aminohexanoate-cyclic-dimer hydrolase |
| 0.00 | 0.02 | 1.01 | -1.34 | 3.49 | 4.89 | K03732 | ATP-dependent RNA helicase RhlB |
| 0.00 | 0.02 | 1.05 | -1.32 | 3.49 | 4.87 | K09895 | uncharacterized protein |
| 0.00 | 0.02 | 1.13 | -1.34 | 3.50 | 4.90 | K15396 | tRNA (cytidine32/uridine32-2'-O)-methyltransferase |
| 0.00 | 0.02 | -2.94 | 1.02 | -8.98 | -11.30 | K01461 | N-acyl-D-glutamate deacylase |
| 0.00 | 0.02 | 1.19 | -1.36 | 3.33 | 4.74 | K04774 | serine protease SohB |
| 0.00 | 0.02 | 1.04 | -1.08 | 1.82 | 2.97 | K10914 | CRP/FNR family transcriptional regulator, cyclic AMP receptor protein |
| 0.00 | 0.02 | 3.10 | 2.27 | 3.96 | 1.74 | K01271 | Xaa-Pro dipeptidase |
| 0.00 | 0.02 | -2.77 | 1.15 | -7.13 | -8.70 | K12989 | mannosyltransferase |
| 0.00 | 0.02 | 1.12 | -1.32 | 3.44 | 4.88 | K03683 | ribonuclease T |
| 0.00 | 0.02 | 0.96 | -1.39 | 3.42 | 4.92 | K03651 | 3',5'-cyclic-AMP phosphodiesterase |
| 0.00 | 0.02 | -1.61 | -0.10 | -3.39 | -3.15 | K09252 | feruloyl esterase |
| 0.00 | 0.02 | 0.76 | -1.37 | 1.57 | 2.96 | K09902 | uncharacterized protein |
| 0.00 | 0.03 | 0.99 | -1.34 | 1.64 | 3.05 | K12297 | 23S rRNA (guanine2069-N7)-methyltransferase / 23S rRNA (guanine2445-N2)-methyltransferase |
| 0.00 | 0.03 | 1.12 | 2.76 | -0.05 | -2.60 | K09009 | uncharacterized protein |
| 0.00 | 0.03 | 0.79 | -1.41 | 1.48 | 2.94 | K07235 | tRNA 2-thiouridine synthesizing protein D |
| 0.00 | 0.03 | 1.20 | 0.03 | 2.37 | 2.22 | K15256 | tRNA (cmo5U34)-methyltransferase |
| 0.00 | 0.03 | -3.79 | -0.15 | -9.53 | -10.32 | K14974 | 6-hydroxynicotinate 3-monooxygenase |
| 0.00 | 0.03 | -2.54 | 1.00 | -7.48 | -9.83 | K08351 | biotin/methionine sulfoxide reductase |
| 0.00 | 0.03 | 1.14 | -0.82 | 3.49 | 4.43 | K07121 | uncharacterized protein |
| 0.00 | 0.03 | 1.22 | 2.99 | 0.41 | -2.63 | K13573 | proteasome accessory factor C |
| 0.00 | 0.03 | -3.97 | -0.19 | -9.15 | -9.90 | K01769 | guanylate cyclase, other |
| 0.00 | 0.03 | -0.32 | 0.85 | -1.08 | -1.97 | K03932 | polyhydroxybutyrate depolymerase |
| 0.00 | 0.03 | -5.54 | -1.09 | -9.01 | -8.04 | K00039 | ribitol 2-dehydrogenase |
| 0.00 | 0.03 | -2.79 | 1.18 | -9.17 | -10.55 | K00082 | 5-amino-6-(5-phosphoribosylamino)uracil reductase |
| 0.00 | 0.03 | -5.29 | -0.76 | -7.71 | -7.00 | K10565 | chemotaxis protein MotD |
| 0.00 | 0.03 | -5.03 | -0.34 | -7.91 | -7.49 | K00875 | D-ribulokinase |
| 0.00 | 0.03 | -2.28 | 1.17 | -7.49 | -8.45 | K16652 | decaprenylphospho-beta-D-erythro-pentofuranosid-2-ulose 2-reductase |
| 0.00 | 0.03 | 0.76 | -1.40 | 1.48 | 2.90 | K07678 | two-component system, NarL family, sensor histidine kinase BarA |
| 0.00 | 0.03 | 3.35 | 1.07 | 4.63 | 3.77 | K00651 | homoserine O-succinyltransferase/O-acetyltransferase |
| 0.00 | 0.03 | 0.93 | -1.32 | 1.49 | 2.94 | K11179 | tRNA 2-thiouridine synthesizing protein E |
| 0.00 | 0.03 | -2.33 | 1.11 | -7.53 | -8.42 | K16653 | decaprenylphospho-beta-D-ribofuranose 2-oxidase |
| 0.00 | 0.03 | 1.25 | 2.99 | 0.58 | -2.41 | K09118 | uncharacterized protein |
| 0.00 | 0.03 | -1.90 | 1.08 | -8.14 | -9.55 | K03396 | S-(hydroxymethyl)glutathione synthase |
| 0.00 | 0.03 | -2.77 | 0.72 | -8.64 | -9.88 | K00493 | xanthocillin biosynthesis cytochrome P450 monooxygenase |
| 0.00 | 0.03 | 0.51 | -2.10 | 3.40 | 5.65 | K10941 | sigma-54 dependent transcriptional regulator, flagellar regulatory protein |
| 0.00 | 0.03 | 1.25 | 3.04 | 0.59 | -2.60 | K07776 | two-component system, OmpR family, response regulator RegX3 |
| 0.00 | 0.03 | 0.40 | -2.03 | 3.32 | 5.53 | K03548 | putative permease |
| 0.00 | 0.03 | -4.10 | -0.27 | -9.38 | -8.87 | K14949 | serine/threonine-protein kinase PknG |
| 0.00 | 0.03 | 0.56 | -2.12 | 3.47 | 5.68 | K19696 | 5'-methylthioinosine phosphorylase |
| 0.00 | 0.03 | 1.94 | 2.88 | -0.25 | -3.17 | K08691 | malyl-CoA/(S)-citramalyl-CoA lyase |
| 0.00 | 0.04 | -2.16 | 1.15 | -7.39 | -8.15 | K14136 | decaprenyl-phosphate phosphoribosyltransferase |
| 0.00 | 0.04 | -1.03 | -3.85 | 1.71 | 5.57 | K00176 | 2-oxoglutarate ferredoxin oxidoreductase subunit delta |
| 0.00 | 0.04 | 0.98 | 3.09 | -0.84 | -3.47 | K08372 | putative serine protease PepD |
| 0.00 | 0.04 | 0.44 | -2.13 | 3.46 | 5.72 | K18800 | 2-polyprenylphenol 6-hydroxylase |
| 0.00 | 0.04 | -1.33 | -7.25 | 0.26 | 8.38 | K07548 | 2-[hydroxy(phenyl)methyl]-succinyl-CoA dehydrogenase BbsD subunit |
| 0.00 | 0.04 | 1.24 | -1.43 | 2.96 | 4.80 | K10943 | two-component system, response regulator FlrC |
| 0.00 | 0.04 | 1.51 | 2.83 | -1.74 | -4.65 | K11159 | carotenoid cleavage oxygenase |
| 0.00 | 0.04 | -0.29 | 1.35 | -1.13 | -2.58 | K13668 | phosphatidyl-myo-inositol dimannoside synthase |
| 0.00 | 0.04 | 0.42 | -2.11 | 3.44 | 5.77 | K16291 | L,D-transpeptidase ErfK/SrfK |
| 0.00 | 0.04 | -6.20 | -1.35 | -9.53 | -8.17 | K14339 | alpha-1,6-mannosyltransferase |
| 0.00 | 0.04 | 1.77 | 0.58 | 2.24 | 1.75 | K09788 | 2-methylaconitate isomerase |
| 0.00 | 0.04 | -1.93 | -7.35 | 0.75 | 7.69 | K09141 | uncharacterized protein |
| 0.00 | 0.04 | -0.47 | -3.50 | 1.19 | 4.95 | K17716 | UDP-glucose 4-epimerase |
| 0.00 | 0.04 | -7.60 | -0.89 | -10.64 | -9.52 | K18028 | 2,5-dihydroxypyridine 5,6-dioxygenase |
| 0.00 | 0.04 | 0.61 | 3.01 | -0.04 | -3.33 | K07768 | two-component system, OmpR family, sensor histidine kinase SenX3 |
| 0.00 | 0.04 | 0.62 | 2.99 | -0.04 | -3.35 | K03727 | ATP-dependent RNA helicase HelY |
| 0.00 | 0.04 | -7.53 | -1.24 | -10.68 | -9.32 | K06021 | universal stress protein A |
| 0.00 | 0.04 | -1.34 | -7.22 | 0.23 | 7.73 | K15019 | 3-hydroxypropionyl-coenzyme A dehydratase |
| 0.00 | 0.04 | -7.62 | -1.27 | -10.59 | -9.37 | K16874 | 2,5-furandicarboxylate decarboxylase 1 |
| 0.00 | 0.04 | 0.64 | 1.72 | 0.08 | -1.72 | K05364 | penicillin-binding protein A |
| 0.00 | 0.04 | -7.52 | -0.98 | -10.52 | -9.28 | K14666 | N-acetylglucosaminyltransferase |
| 0.00 | 0.04 | 0.56 | -2.03 | 1.72 | 4.04 | K09920 | uncharacterized protein |
| 0.00 | 0.04 | 0.29 | -2.21 | 1.62 | 3.87 | K07637 | two-component system, OmpR family, sensor histidine kinase PhoQ |
| 0.00 | 0.04 | -2.28 | -5.02 | -0.37 | 4.71 | K13694 | murein DD-endopeptidase / murein LD-carboxypeptidase |
| 0.00 | 0.04 | -1.03 | -3.85 | 1.67 | 5.70 | K00177 | 2-oxoglutarate ferredoxin oxidoreductase subunit gamma |
| 0.00 | 0.04 | 0.71 | -0.86 | 1.67 | 2.55 | K09912 | uncharacterized protein |
| 0.00 | 0.04 | 2.34 | 1.80 | 3.60 | 1.95 | K09790 | uncharacterized protein |
| 0.00 | 0.04 | -2.30 | -7.14 | -0.15 | 6.95 | K00288 | methylenetetrahydrofolate dehydrogenase (NADP+) / methenyltetrahydrofolate cyclohydrolase / formyltetrahydrofolate synthetase |
| 0.00 | 0.04 | -1.35 | 1.31 | -3.85 | -4.50 | K08253 | non-specific protein-tyrosine kinase |
| 0.01 | 0.04 | -7.49 | -1.19 | -10.64 | -8.93 | K14633 | ketoreductase RED2 |
| 0.00 | 0.04 | -2.33 | -6.35 | -0.03 | 6.29 | K03388 | heterodisulfide reductase subunit A2 |
| 0.00 | 0.04 | 3.88 | 4.50 | 2.64 | -1.84 | K05794 | tellurite resistance protein TerC |
| 0.00 | 0.04 | -7.24 | -0.88 | -10.01 | -9.01 | K14657 | LysR family transcriptional regulator, nod-box dependent transcriptional activator |
| 0.00 | 0.04 | 1.56 | 3.25 | -0.32 | -3.66 | K01796 | alpha-methylacyl-CoA racemase |
| 0.01 | 0.05 | -7.36 | -0.95 | -10.57 | -9.46 | K19731 | LuxR family transcriptional regulator, quorum-sensing system regulator CciR |
| 0.01 | 0.05 | -7.51 | -1.14 | -10.61 | -9.12 | K13028 | aliphatic aldoxime dehydratase |
| 0.00 | 0.05 | -1.60 | 1.52 | -3.25 | -4.85 | K08166 | MFS transporter, DHA2 family, methylenomycin A resistance protein |
| 0.00 | 0.05 | 0.33 | -2.08 | 1.77 | 4.03 | K00569 | thiopurine S-methyltransferase |
| 0.01 | 0.05 | -7.47 | -0.98 | -10.60 | -9.49 | K14658 | nodulation protein A |
| 0.01 | 0.05 | -7.55 | -1.24 | -10.61 | -9.31 | K14659 | chitooligosaccharide deacetylase |
| 0.00 | 0.05 | 0.34 | -2.00 | 1.64 | 3.83 | K07660 | two-component system, OmpR family, response regulator PhoP |
| 0.00 | 0.05 | -2.26 | -7.14 | -0.12 | 6.91 | K00087 | xanthine dehydrogenase molybdenum-binding subunit |
| 0.00 | 0.05 | 0.29 | -2.11 | 1.74 | 3.92 | K06149 | universal stress protein A |
| 0.00 | 0.05 | -1.54 | -3.35 | 0.48 | 3.69 | K01630 | 2-dehydro-3-deoxyglucarate aldolase |
| 0.00 | 0.05 | -1.23 | 1.05 | -3.82 | -4.78 | K02568 | nitrate reductase (cytochrome), electron transfer subunit |

**Supplementary Table 8** Export of differences underlined with the statistical comparison of gorge and cave samples PWY abundances.

|  | **glm.ep** | **glm.eBH** | **rab.all** | **rab.win.Forra** | **rab.win.Grotta** | **diff.btw** | **PWY** | **Name** |
| --- | --- | --- | --- | --- | --- | --- | --- | --- |
| a | 0.00 | 0.04 | -3.82 | -0.50 | -11.67 | -11.50 | P621-PWY | nylon-6 oligomer degradation |
| b | 0.00 | 0.04 | -1.08 | 0.98 | -2.68 | -3.97 | PWY-6876 | isopropanol biosynthesis |

**Supplementary Table 9** Primer 7 software output obtained for principal component analysis.

| **Eigenvalues** | | | | | |
| --- | --- | --- | --- | --- | --- |
| PC | Eigenvalues | %Variation | Cum.%Variation | |  |
| 1 | 17.6 | 36.0 | 36.0 |  |  |
| 2 | 13 | 26.6 | 62.6 |  |  |
| 3 | 5.52 | 11.3 | 73.8 |  |  |
| 4 | 5.09 | 10.4 | 84.2 |  |  |
| 5 | 3.96 | 8.1 | 92.3 |  |  |
|  | |  |  |  |  |
| **Eigenvectors** | | | | | |
| Variable | PC1 | PC2 | PC3 | PC4 | PC5 |
| Taxa_S | -0.147 | 0.097 | -0.036 | 0.282 | 0.104 |
| Chao-1 | -0.148 | 0.096 | -0.037 | 0.281 | 0.103 |
| Shannon_H | -0.102 | 0.181 | -0.156 | 0.221 | -0.011 |
| Simpson_1-D | -0.074 | 0.199 | -0.178 | 0.183 | -0.087 |
| pH | -0.177 | -0.132 | 0.198 | 0.020 | 0.000 |
| H % | -0.177 | -0.132 | 0.198 | 0.020 | 0.000 |
| T | 0.177 | 0.132 | -0.198 | -0.020 | 0.000 |
| Acidobacteriota | 0.095 | 0.060 | 0.023 | 0.351 | 0.201 |
| Actinobacteriota | 0.134 | -0.045 | -0.182 | 0.164 | -0.246 |
| Bacteroidota | -0.077 | 0.116 | -0.153 | -0.283 | 0.121 |
| Chloroflexota | 0.060 | -0.101 | -0.009 | 0.289 | 0.085 |
| Crenarchaeota | 0.202 | -0.074 | 0.123 | -0.051 | 0.149 |
| BacillotaBacillota | -0.130 | -0.215 | -0.060 | -0.110 | 0.025 |
| Gemmatimonadota | -0.055 | 0.113 | -0.228 | 0.044 | 0.301 |
| Methylomirabilota | -0.120 | -0.016 | 0.156 | 0.220 | 0.113 |
| Micrarchaeota | 0.202 | -0.074 | 0.123 | -0.051 | 0.149 |
| Myxococcota | -0.004 | 0.209 | -0.194 | -0.147 | 0.150 |
| Nitrospirota | -0.076 | 0.215 | 0.164 | -0.073 | 0.025 |
| Patescibacteria | 0.203 | 0.031 | 0.042 | -0.039 | -0.106 |
| Planctomycetota | -0.170 | 0.004 | 0.200 | 0.108 | 0.159 |
| Pseudomonadota | -0.148 | 0.138 | 0.090 | -0.232 | -0.085 |
| Thermoplasmatota | 0.202 | -0.074 | 0.123 | -0.051 | 0.149 |
| Verrucomicrobiota | 0.043 | 0.187 | -0.276 | -0.024 | -0.149 |
| Glyceraldehyde-3-P dehydrogenase | -0.187 | -0.075 | 0.067 | 0.022 | 0.208 |
| Pyruvate dehydrogenase (lipoamide) | 0.021 | -0.228 | -0.090 | 0.141 | -0.166 |
| Succinate dehydrogenase (ubiquinone) | -0.088 | 0.245 | 0.022 | 0.096 | -0.044 |
| Nitrite reductase (NADH) | 0.117 | 0.134 | 0.035 | -0.188 | 0.145 |
| Nitrate reductase | 0.200 | 0.024 | -0.115 | 0.135 | 0.099 |
| Dihydrolipoyl dehydrogenase | -0.230 | -0.052 | -0.060 | -0.029 | -0.020 |
| Protein-disulfide reductase | -0.061 | 0.161 | 0.150 | -0.085 | -0.079 |
| Thioredoxin-disulfide reductase | -0.027 | -0.266 | -0.080 | 0.024 | -0.050 |
| Peptide-methionine (S)-S-oxide reductase | 0.089 | -0.032 | -0.029 | -0.168 | 0.390 |
| Peptide-methionine (R)-S-oxide reductase | 0.080 | 0.189 | 0.073 | -0.106 | 0.229 |
| Cytochrome-c oxidase | 0.223 | -0.014 | 0.118 | 0.061 | -0.081 |
| Peroxiredoxin | -0.191 | 0.153 | -0.021 | -0.074 | 0.029 |
| Superoxide dismutase | -0.137 | -0.154 | -0.221 | -0.112 | 0.021 |
| Arsenate reductase (glutaredoxin) | -0.184 | 0.117 | -0.142 | 0.036 | -0.127 |
| uroporphyrin-III C-methyltransferase | -0.199 | 0.006 | 0.224 | -0.025 | -0.061 |
| transketolase (mechanism) | -0.162 | -0.117 | -0.217 | -0.109 | 0.087 |
| citrate (Si)-synthase | 0.136 | 0.213 | 0.085 | 0.039 | -0.046 |
| 6-Phosphofructokinase | -0.153 | 0.074 | -0.126 | -0.098 | 0.230 |
| Cd2+ exporting ATPase | -0.118 | -0.211 | -0.133 | -0.067 | 0.088 |
| fructose-bisphosphate aldolase (mechanism) | -0.019 | -0.231 | 0.007 | 0.149 | 0.173 |
| Fumarate hydratase | -0.161 | 0.088 | 0.233 | 0.152 | 0.019 |
| Aconitate hydratase | -0.055 | 0.162 | 0.065 | 0.112 | 0.267 |
| sirohydrochlorin ferrochelatase | -0.120 | -0.209 | -0.133 | -0.039 | 0.098 |
| ribose-5-phosphate isomerase | -0.188 | -0.077 | 0.090 | -0.140 | -0.065 |
| glucose-6-phosphate isomerase | -0.076 | 0.149 | 0.252 | -0.124 | -0.188 |
| cobaltochelatase | 0.202 | -0.074 | 0.123 | -0.051 | 0.149 |
|  |  |  |  |  |  |
| **Principal Component Scores** | | | | | |
| Sample | SCORE1 | SCORE2 | SCORE3 | SCORE4 | SCORE5 |
| G1 | 8.8 | -2.39 | 1.68 | -0.647 | 1.47 |
| G2 | -0.0898 | 4.34 | -2.18 | 0.522 | 3.1 |
| G3 | -0.125 | 4.53 | -1.24 | -3.15 | -1.41 |
| G4 | 3.08 | -0.0282 | -2.36 | 2.9 | -3.16 |
| C1 | -3.04 | -5.21 | -1.3 | -3.02 | -0.0467 |
| C2 | -3.82 | -3.6 | -1.07 | 2.37 | 1.3 |
| C3 | -3.13 | 2.47 | 3.53 | 1.35 | 0.242 |
| C4 | -1.67 | -0.107 | 2.94 | -0.333 | -1.5 |

**Supplementary Table 10** Environmental parameters obtained from the sampling sites.

|  | **G1** | **G2** | **G3** | | **G4** | **C1** | **C2** | **C3** | **C4** |
| --- | --- | --- | --- | --- | --- | --- | --- | --- | --- |
| **Source** | Gorge | | | | | Cave | | | |
| **Site** | Infernaccio | | | | | Grotta Grande dei Cervi | | Ovito di Pietrasecca | |
| **Consistency** | Corroded | Solid | Corroded | | Solid | Solid | Solid | Solid | Solid |
| **Environment** | Epigean | Epigean | Epigean | | Epigean | Hypogean | Hypogean | Hypogean | Hypogean |
| **pH** | 6.7 | 6.7 | 6.7 | | 6.7 | 7.1 | 7.1 | 7.1 | 7.1 |
| **Humidity** | 60 | 60 | 60 | | 60 | 98 | 98 | 98 | 98 |
| **Temperature** | 18 | 18 | 18 | | 18 | 8 | 8 | 8 | 8 |
| **State of activity** | Active | | | | | Not active | | Active | |
|  | **Sequencing results** | | | | | | | | |
| **non-chimeric amplicons** | 41352 | 28169 | | 21297 | 35246 | 24417 | 52407 | 55588 | 25207 |

**Supplementary Table 11** List of EC involved with redox biotransformations, metal resistance, and respiratory processes used for Mn metabolism search in EC PICRUSt export.

| **EC Number** | **Description** | **Detail** |
| --- | --- | --- |
| 1.1.1.37 | Malate dehydrogenase | Glycolysis/TCA cycle - aerobic respiration |
| 1.1.1.41 | isocitrate dehydrogenase (NAD+) | Glycolysis/TCA cycle - aerobic respiration |
| 1.1.1.42 | isocitrate dehydrogenase (NADP+) | Glycolysis/TCA cycle - aerobic respiration |
| 1.1.2.2 | Mannitol dehydrogenase (cytochrome) | Cytochrome oxidoreductase |
| 1.1.2.3 | L-lactate dehydrogenase (cytochrome) | Cytochrome oxidoreductase |
| 1.1.2.4 | D-lactate dehydrogenase (cytochrome) | Cytochrome oxidoreductase |
| 1.1.2.5 | D-lactate dehydrogenase (cytochrome c-553) | Cytochrome oxidoreductase |
| 1.1.2.6 | Polyvinyl alcohol dehydrogenase (cytochrome) | Cytochrome oxidoreductase |
| 1.1.2.7 | Methanol dehydrogenase (cytochrome c) | Cytochrome oxidoreductase |
| 1.1.2.8 | Alcohol dehydrogenase (cytochrome c) | Cytochrome oxidoreductase |
| 1.1.2.9 | 1-butanol dehydrogenase (cytochrome c) | Cytochrome oxidoreductase |
| 1.2.1.12 | Glyceraldehyde-3-P dehydrogenase | Glycolysis/TCA cycle - aerobic respiration |
| 1.2.1.13 | glyceraldehyde-3-phosphate dehydrogenase (NADP ) + (phosphorylating) | Autotrophy / Calvin cycle autotrophy & also used in heterotrophy |
| 1.2.1.16 | succinate-semialdehyde dehydrogenase [NAD(P)+] | Glycolysis/TCA cycle - aerobic respiration |
| 1.2.1.24 | succinate-semialdehyde dehydrogenase (NAD+) | Glycolysis/TCA cycle - aerobic respiration |
| 1.2.1.79 | succinate-semialdehyde dehydrogenase (NADP+) | Glycolysis/TCA cycle - aerobic respiration |
| 1.2.2.1 | Formate dehydrogenase (cytochrome) | Cytochrome oxidoreductase |
| 1.2.2.4 | Carbon-monoxide dehydrogenase (cytochrome b-561) | Cytochrome oxidoreductase |
| 1.2.4.1 | Pyruvate dehydrogenase (lipoamide) | Glycolysis/TCA cycle - aerobic respiration |
| 1.2.4.2 | Oxoglutarate dehydrogenase (lipoamide/succinyl-transferring) | Glycolysis/TCA cycle - aerobic respiration |
| 1.3.1.54 | precorrin-6X reductase | Cobalamin biosynthesis |
| 1.3.1.76 | precorrin-2 dehydrogenase | Cobalamin biosynthesis |
| 1.3.2.3 | L-galactonolactone dehydrogenase | Cytochrome oxidoreductase |
| 1.3.5.1 | Succinate dehydrogenase (ubiquinone) | Glycolysis/TCA cycle - aerobic respiration |
| 1.4.2.1 | Glycine dehydrogenase (cytochrome) | Cytochrome oxidoreductase |
| 1.7.1.1 | Nitrate reductase (NADH) | Nitrogen compound oxidoreductase |
| 1.7.1.10 | Hydroxylamine reductase (NADH) | Nitrogen compound oxidoreductase |
| 1.7.1.11 | 4-(dimethylamino)phenylazoxybenzene reductase | Nitrogen compound oxidoreductase |
| 1.7.1.12 | N-hydroxy-2-acetamidofluorene reductase | Nitrogen compound oxidoreductase |
| 1.7.1.13 | PreQ(1) synthase | Nitrogen compound oxidoreductase |
| 1.7.1.14 | Nitric oxide reductase (NAD(P)(+), nitrous oxide-forming) | Nitrogen compound oxidoreductase |
| 1.7.1.15 | Nitrite reductase (NADH) | Nitrogen compound oxidoreductase |
| 1.7.1.16 | Nitrobenzene nitroreductase | Nitrogen compound oxidoreductase |
| 1.7.1.17 | FMN-dependent NADH-azoreductase | Nitrogen compound oxidoreductase |
| 1.7.1.2 | Nitrate reductase (NAD(P)H) | Nitrogen compound oxidoreductase |
| 1.7.1.3 | Nitrate reductase (NADPH) | Nitrogen compound oxidoreductase |
| 1.7.1.4 | Nitrite reductase (NAD(P)H) | Nitrogen compound oxidoreductase / Nitrite oxidation |
| 1.7.1.5 | Hyponitrite reductase | Nitrogen compound oxidoreductase |
| 1.7.1.6 | Azobenzene reductase | Nitrogen compound oxidoreductase |
| 1.7.1.7 | GMP reductase | Nitrogen compound oxidoreductase |
| 1.7.1.9 | Nitroquinoline-N-oxide reductase | Nitrogen compound oxidoreductase |
| 1.7.2.1 | Nitrite reductase (NO-forming) | Nitrogen compound oxidoreductase / Cytochrome oxidoreductase |
| 1.7.2.2 | Nitrite reductase (cytochrome; ammonia-forming) | Nitrogen compound oxidoreductase / Cytochrome oxidoreductase |
| 1.7.2.3 | Trimethylamine-N-oxide reductase | Nitrogen compound oxidoreductase / Cytochrome oxidoreductase |
| 1.7.2.4 | Nitrous-oxide reductase | Nitrogen compound oxidoreductase / Cytochrome oxidoreductase |
| 1.7.2.5 | Nitric-oxide reductase (cytochrome c) | Nitrogen compound oxidoreductase / Cytochrome oxidoreductase |
| 1.7.2.6 | Hydroxylamine dehydrogenase | Nitrogen compound oxidoreductase / Cytochrome oxidoreductase / Ammonia oxidation |
| 1.7.2.7 | Hydrazine synthase | Nitrogen compound oxidoreductase / Cytochrome oxidoreductase |
| 1.7.2.8 | Hydrazine dehydrogenase | Nitrogen compound oxidoreductase / Cytochrome oxidoreductase |
| 1.7.3.1 | Nitroalkane oxidase | Nitrogen compound oxidoreductase |
| 1.7.3.2 | Acetylindoxyl oxidase | Nitrogen compound oxidoreductase |
| 1.7.3.3 | Factor independent urate hydroxylase | Nitrogen compound oxidoreductase |
| 1.7.3.5 | 3-aci-nitropropanoate oxidase | Nitrogen compound oxidoreductase |
| 1.7.3.6 | Hydroxylamine oxidase (cytochrome) | Nitrogen compound oxidoreductase |
| 1.7.5.1 | Nitrate reductase (quinone) | Nitrogen compound oxidoreductase |
| 1.7.5.2 | Nitric oxide reductase (menaquinol) | Nitrogen compound oxidoreductase |
| 1.7.6.1 | Nitrite dismutase | Nitrogen compound oxidoreductase |
| 1.7.7.1 | Ferredoxin--nitrite reductase | Nitrogen compound oxidoreductase |
| 1.7.7.2 | Ferredoxin--nitrate reductase | Nitrogen compound oxidoreductase |
| 1.7.99.1 | Hydroxylamine reductase | Nitrogen compound oxidoreductase |
| 1.7.99.4 | Nitrate reductase | Nitrogen compound oxidoreductase |
| 1.8.1.10 | CoA-glutathione reductase | Sulphur group oxidoreductase |
| 1.8.1.11 | Asparagusate reductase | Sulphur group oxidoreductase |
| 1.8.1.12 | Trypanothione-disulfide reductase | Sulphur group oxidoreductase |
| 1.8.1.13 | Bis-gamma-glutamylcystine reductase | Sulphur group oxidoreductase |
| 1.8.1.14 | CoA-disulfide reductase | Sulphur group oxidoreductase |
| 1.8.1.15 | Mycothione reductase | Sulphur group oxidoreductase |
| 1.8.1.16 | Glutathione amide reductase | Sulphur group oxidoreductase |
| 1.8.1.17 | Dimethylsulfone reductase | Sulphur group oxidoreductase |
| 1.8.1.18 | NAD(P)H Sulphur oxidoreductase (CoA-dependent) | Sulphur group oxidoreductase |
| 1.8.1.19 | Sulfide dehydrogenase | Sulphur group oxidoreductase |
| 1.8.1.2 | Assimilatory sulfite reductase (NADPH) | Sulphur group oxidoreductase |
| 1.8.1.20 | 4,4'-dithiodibutanoate disulfide reductase | Sulphur group oxidoreductase |
| 1.8.1.21 | Dissimilatory dimethyldisulfide reductase | Sulphur group oxidoreductase |
| 1.8.1.3 | Hypotaurine dehydrogenase | Sulphur group oxidoreductase |
| 1.8.1.4 | Dihydrolipoyl dehydrogenase | Sulphur group oxidoreductase / Glycolysis/TCA cycle - aerobic respiration |
| 1.8.1.5 | 2-oxopropyl-CoM reductase (carboxylating) | Sulphur group oxidoreductase |
| 1.8.1.6 | Cystine reductase | Sulphur group oxidoreductase |
| 1.8.1.7 | Glutathione-disulfide reductase | Sulphur group oxidoreductase |
| 1.8.1.8 | Protein-disulfide reductase | Sulphur group oxidoreductase |
| 1.8.1.9 | Thioredoxin-disulfide reductase | Sulphur group oxidoreductase |
| 1.8.2.1 | Sulfite dehydrogenase (cytochrome) | Sulphur group oxidoreductase / Cytochrome oxidoreductase |
| 1.8.2.2 | Thiosulfate dehydrogenase | Cytochrome oxidoreductase |
| 1.8.2.3 | Sulfide-cytochrome-c reductase (flavocytochrome c) | Sulphur group oxidoreductase / Cytochrome oxidoreductase |
| 1.8.2.4 | Dimethyl sulfide:cytochrome c2 reductase | Sulphur group oxidoreductase / Cytochrome oxidoreductase |
| 1.8.2.5 | Thiosulfate reductase (cytochrome) | Sulphur group oxidoreductase / Cytochrome oxidoreductase |
| 1.8.2.6 | S-disulfanyl-L-cysteine oxidoreductase | Sulphur group oxidoreductase / Cytochrome oxidoreductase |
| 1.8.3.1 | Sulfite oxidase | Sulphur group oxidoreductase |
| 1.8.3.2 | Thiol oxidase | Sulphur group oxidoreductase |
| 1.8.3.3 | Glutathione oxidase | Sulphur group oxidoreductase |
| 1.8.3.4 | Methanethiol oxidase | Sulphur group oxidoreductase |
| 1.8.3.5 | Prenylcysteine oxidase | Sulphur group oxidoreductase |
| 1.8.3.6 | Farnesylcysteine lyase | Sulphur group oxidoreductase |
| 1.8.3.7 | Formylglycine-generating enzyme | Sulphur group oxidoreductase |
| 1.8.4.1 | Glutathione--homocystine transhydrogenase | Sulphur group oxidoreductase |
| 1.8.4.10 | Adenylyl-sulfate reductase (thioredoxin) | Sulphur group oxidoreductase |
| 1.8.4.11 | Peptide-methionine (S)-S-oxide reductase | Sulphur group oxidoreductase |
| 1.8.4.12 | Peptide-methionine (R)-S-oxide reductase | Sulphur group oxidoreductase |
| 1.8.4.13 | L-methionine (S)-S-oxide reductase | Sulphur group oxidoreductase |
| 1.8.4.14 | L-methionine (R)-S-oxide reductase | Sulphur group oxidoreductase |
| 1.8.4.2 | Protein-disulfide reductase (glutathione) | Sulphur group oxidoreductase |
| 1.8.4.3 | Glutathione--CoA-glutathione transhydrogenase | Sulphur group oxidoreductase |
| 1.8.4.4 | Glutathione--cystine transhydrogenase | Sulphur group oxidoreductase |
| 1.8.4.7 | Enzyme-thiol transhydrogenase (glutathione-disulfide) | Sulphur group oxidoreductase |
| 1.8.4.8 | Phosphoadenylyl-sulfate reductase (thioredoxin) | Sulphur group oxidoreductase |
| 1.8.4.9 | Adenylyl-sulfate reductase (glutathione) | Sulphur group oxidoreductase |
| 1.8.5.1 | Glutathione dehydrogenase (ascorbate) | Sulphur group oxidoreductase |
| 1.8.5.2 | Thiosulfate dehydrogenase (quinone) | Sulphur group oxidoreductase |
| 1.8.5.3 | Respiratory dimethylsulfoxide reductase | Sulphur group oxidoreductase |
| 1.8.5.4 | Bacterial sulfide:quinone reductase | Sulphur group oxidoreductase |
| 1.8.5.5 | Thiosulfate reductase (quinone) | Sulphur group oxidoreductase |
| 1.8.5.6 | Sulfite dehydrogenase (quinone) | Sulphur group oxidoreductase |
| 1.8.5.7 | Glutathionyl-hydroquinone reductase | Sulphur group oxidoreductase |
| 1.8.5.8 | Eukaryotic sulfide quinone oxidoreductase | Sulphur group oxidoreductase |
| 1.8.7.1 | Assimilatory sulfite reductase (ferredoxin) | Sulphur group oxidoreductase |
| 1.8.7.2 | Ferredoxin:thioredoxin reductase | Sulphur group oxidoreductase |
| 1.8.7.3 | Ferredoxin:CoB-CoM heterodisulfide reductase | Sulphur group oxidoreductase |
| 1.8.98.1 | Dihydromethanophenazine:CoB--CoM heterodisulfide reductase | Sulphur group oxidoreductase |
| 1.8.98.2 | Sulfiredoxin | Sulphur group oxidoreductase |
| 1.8.98.3 | Sulfite reductase (coenzyme F420) | Sulphur group oxidoreductase |
| 1.8.98.4 | Coenzyme F420:CoB-CoM heterodisulfide,ferredoxin reductase | Sulphur group oxidoreductase |
| 1.8.98.5 | H(2):CoB-CoM heterodisulfide,ferredoxin reductase | Sulphur group oxidoreductase |
| 1.8.98.6 | Formate:CoB-CoM heterodisulfide,ferredoxin reductase | Sulphur group oxidoreductase |
| 1.8.99.2 | Adenylyl-sulfate reductase | Sulphur group oxidoreductase |
| 1.8.99.5 | Dissimilatory sulfite reductase | Sulphur group oxidoreductase |
| 1.9.3.1 | Cytochrome-c oxidase | Heme group oxidoreductase |
| 1.9.6.1 | Nitrate reductase (cytochrome) | Heme group oxidoreductase |
| 1.9.98.1 | Iron--cytochrome-c reductase | Heme group oxidoreductase |
| 1.10.2.1 | L-ascorbate--cytochrome-b5 reductase | Cytochrome oxidoreductase |
| 1.11.1.13 | Manganese peroxidase | Mn/Fe cycling |
| 1.11.1.15 | Peroxiredoxin | Metal resistance/transport |
| 1.11.1.19 | Glutathione peroxidase | Metal resistance/transport |
| 1.12.1.2 | Hydrogen dehydrogenase | Hydogenase |
| 1.12.1.3 | Hydrogen dehydrogenase (NADP(+)) | Hydogenase |
| 1.12.1.4 | Hydrogenase (NAD(+), ferredoxin) | Hydogenase |
| 1.12.1.5 | Hydrogen dehydrogenase (NAD(P)(+)) | Hydogenase |
| 1.12.2.1 | Cytochrome-c3 hydrogenase | Hydogenase, cytochrome oxidoreductase, Mn/Fe cycling |
| 1.12.5.1 | Hydrogen:quinone oxidoreductase | Hydogenase |
| 1.12.7.2 | Ferredoxin hydrogenase | Hydogenase |
| 1.12.98.1 | Coenzyme F420 hydrogenase | Hydogenase |
| 1.12.98.2 | 5,10-methenyltetrahydromethanopterin hydrogenase | Hydogenase |
| 1.12.98.3 | Methanosarcina-phenazine hydrogenase | Hydogenase |
| 1.12.98.4 | Sulfhydrogenase | Hydogenase |
| 1.12.99.6 | Hydrogenase (acceptor) | Hydogenase |
| 1.13.11.15 | Sulphur oxygenase/reductase | S cycling |
| 1.14.13.83 | precorrin-3B synthase (mechanism) | Cobalamin biosynthesis |
| 1.14.99.39 | Ammonia monooxygenase | Ammonia oxidation |
| 1.15.1.1 | Superoxide dismutase | Superoxide /Metal resistance/transport |
| 1.15.1.2 | Superoxide reductase | Superoxide |
| 1.16.1.1 | Mercury(II) reductase | Metal oxidoreductase |
| 1.16.1.10 | Ferric-chelate reductase (NAD(P)H) | Metal oxidoreductase |
| 1.16.1.2 | Diferric-transferrin reductase | Metal oxidoreductase |
| 1.16.1.3 | Aquacobalamin reductase | Metal oxidoreductase |
| 1.16.1.4 | Cob(II)alamin reductase | Metal oxidoreductase |
| 1.16.1.5 | Aquacobalamin reductase (NADPH) | Metal oxidoreductase |
| 1.16.1.6 | Cyanocobalamin reductase (cyanide-eliminating) | Metal oxidoreductase |
| 1.16.1.7 | Ferric-chelate reductase (NADH) | Metal oxidoreductase |
| 1.16.1.8 | [Methionine synthase] reductase | Metal oxidoreductase |
| 1.16.1.9 | Ferric-chelate reductase (NADPH) | Metal oxidoreductase |
| 1.16.3.1 | Ferroxidase | Metal oxidoreductase / Mn/Fe cycling |
| 1.16.3.2 | Bacterial non-heme ferritin | Metal oxidoreductase / Mn/Fe cycling |
| 1.16.3.3 | Manganese oxidase | Metal oxidoreductase |
| 1.16.8.1 | Cob(II)yrinic acid a,c-diamide reductase | Metal oxidoreductase / Cobalamin biosynthesis |
| 1.16.9.1 | Iron:rusticyanin reductase | Metal oxidoreductase |
| 1.17.2.1 | Nicotinate dehydrogenase (cytochrome) | Cytochrome oxidoreductase |
| 1.17.2.2 | Lupanine 17-hydroxylase (cytochrome c) | Cytochrome oxidoreductase |
| 1.17.2.3 | Formate dehydrogenase (cytochrome c-553) | Cytochrome oxidoreductase |
| 1.20.1.1 | Phosphonate dehydrogenase | As or P oxidoreductase |
| 1.20.2.1 | Arsenate reductase (cytochrome c) | Cytochrome oxidoreductase / As or P oxidoreductase |
| 1.20.4.1 | Arsenate reductase (glutaredoxin) | As or P oxidoreductase |
| 1.20.4.2 | Methylarsonate reductase | As or P oxidoreductase |
| 1.20.4.3 | Mycoredoxin | As or P oxidoreductase |
| 1.20.4.4 | Arsenate reductase (thioredoxin) | As or P oxidoreductase |
| 1.20.9.1 | Arsenate reductase (azurin) | As or P oxidoreductase |
| 1.20.99.1 | Arsenate reductase (donor) | As or P oxidoreductase |
| 1.97.1.12 | Photosystem I | Other oxidoreductase |
| 1.97.1.9 | Selenate reductase | Other oxidoreductase |
| 2.1.1.107 | uroporphyrin-III C-methyltransferase | Cobalamin biosynthesis |
| 2.1.1.130 | precorrin-2 C20-methyltransferase | Cobalamin biosynthesis |
| 2.1.1.131 | precorrin-3B C17-methyltransferase (mechanism) | Cobalamin biosynthesis |
| 2.1.1.132 | precorrin-6B C5,15-methyltransferase (decarboxylating) (mechanism) | Cobalamin biosynthesis |
| 2.1.1.133 | precorrin-4 C11-methyltransferase | Cobalamin biosynthesis |
| 2.1.1.151 | cobalt-factor II C20-methyltransferase | Cobalamin biosynthesis |
| 2.1.1.152 | precorrin-5 C1-methyltransferase (deacetylating) (mechanism) | Cobalamin biosynthesis |
| 2.1.1.195 | cobalt-precorrin-5B (C1)-methyltransferase | Cobalamin biosynthesis |
| 2.1.1.271 | cobalt-precorrin-4 methyltransferase | Cobalamin biosynthesis |
| 2.1.1.272 | cobalt-factor III methyltransferase | Cobalamin biosynthesis |
| 2.2.1.1 | transketolase (mechanism) | Calvin cycle autotrophy & also used in heterotrophy |
| 2.3.1.61 | dihydrolipoyllysine-residue succinyltransferase | Glycolysis/TCA cycle - aerobic respiration |
| 2.3.3.1 | citrate (Si)-synthase | Glycolysis/TCA cycle - aerobic respiration |
| 2.7.1.1 | Hexokinase | Glycolysis/TCA cycle - aerobic respiration |
| 2.7.1.11 | 6-Phosphofructokinase | Glycolysis/TCA cycle - aerobic respiration |
| 2.7.1.146 | ADP-specific phosphofructokinase | Glycolysis/TCA cycle - aerobic respiration |
| 2.7.1.156 | adenosylcobinamide kinase | Cobalamin biosynthesis |
| 2.7.1.177 | L-threonine kinase | Cobalamin biosynthesis |
| 2.7.1.19 | phosphoribulokinase | Calvin cycle autotrophy & also used in heterotrophy |
| 2.7.1.40 | Pyruvate kinase | Glycolysis/TCA cycle - aerobic respiration |
| 2.7.1.90 | diphosphate—fructose-6-phosphate 1-phosphotransferase | Glycolysis/TCA cycle - aerobic respiration |
| 2.7.2.3 | phosphoglycerate kinase | Autotrophy / Calvin cycle autotrophy & also used in heterotrophy |
| 2.7.7.62 | adenosylcobinamide phosphate guanylyltransferase | Cobalamin biosynthesis |
| 3.1.2.3 | Succinyl-CoA hydrolase | Glycolysis/TCA cycle - aerobic respiration |
| 3.1.3.11 | fructose-bisphosphatase | Calvin cycle autotrophy & also used in heterotrophy |
| 3.1.3.37 | sedoheptulose-bisphosphatase | Calvin cycle autotrophy & also used in heterotrophy |
| 3.6.3.3 | Cd2+ exporting ATPase | Metal resistance/transport |
| 3.7.1.12 | cobalt-precorrin 5A hydrolase | Cobalamin biosynthesis |
| 4.1.1.39 | ribulose-bisphosphate carboxylase | Autotrophy / Calvin cycle autotrophy & also used in heterotrophy |
| 4.1.1.71 | 2-oxoglutarate decarboxylase | Glycolysis/TCA cycle - aerobic respiration |
| 4.1.1.81 | threonine phosphate decarboxylase | Cobalamin biosynthesis |
| 4.1.2.13 | fructose-bisphosphate aldolase (mechanism) | Calvin cycle autotrophy & also used in heterotrophy |
| 4.1.3.7 | Citrate synthase | Glycolysis/TCA cycle - aerobic respiration |
| 4.2.1.11 | Phosphopyruvate hydratase | Glycolysis/TCA cycle - aerobic respiration |
| 4.2.1.2 | Fumarate hydratase | Glycolysis/TCA cycle - aerobic respiration |
| 4.2.1.3 | Aconitate hydratase | Glycolysis/TCA cycle - aerobic respiration |
| 4.99.1.3 | sirohydrochlorin cobaltochelatase | Cobalamin biosynthesis |
| 4.99.1.4 | sirohydrochlorin ferrochelatase | Cobalamin biosynthesis |
| 5.1.3.1 | ribulose-phosphate 3-epimerase | Calvin cycle autotrophy & also used in heterotrophy |
| 5.3.1.1 | triose-phosphate isomerase | Calvin cycle autotrophy & also used in heterotrophy |
| 5.3.1.6 | ribose-5-phosphate isomerase | Calvin cycle autotrophy & also used in heterotrophy |
| 5.3.1.9 | glucose-6-phosphate isomerase | Calvin cycle autotrophy & also used in heterotrophy |
| 5.4.2.1 | Phosphoglycerate mutase | Glycolysis/TCA cycle - aerobic respiration |
| 5.4.99.61 | precorrin-8X methylmutase (formerly 5.4.1.2) | Cobalamin biosynthesis |
| 6.2.1.4 | Succinate-CoA ligase | Glycolysis/TCA cycle - aerobic respiration |
| 6.3.1.10 | adenosylcobinamide phosphate synthase | Cobalamin biosynthesis |
| 6.3.5.9 | hydrogenobyrinic acid a,c-diamide synthase (glutamine- hydrolysing) | Cobalamin biosynthesis |
| 6.6.1.2 | cobaltochelatase | Cobalamin biosynthesis |
| 7.1.1.7 | Ubiquinol oxidase (electrogenic, proton-motive force generating) | Metal resistance/transport |
| 7.2.1.1 | NADH:ubiquinone reductase (Na(+)-transporting) | Metal resistance/transport |
| 7.2.1.2 | Ferredoxin--NAD(+) oxidoreductase (Na(+)-transporting) | Metal resistance/transport |
| 7.2.1.3 | Ascorbate ferrireductase (transmembrane) | Metal resistance/transport |
| 7.2.2.1 | Na(+)-transporting two-sector ATPase | Metal resistance/transport |
| 7.2.2.10 | P-type Ca(2+) transporter | Metal resistance/transport |
| 7.2.2.11 | ABC-type Ni(2+) transporter | Metal resistance/transport |
| 7.2.2.12 | P-type Zn(2+) transporter | Metal resistance/transport |
| 7.2.2.13 | Na(+)/K(+)-exchanging ATPase | Metal resistance/transport |
| 7.2.2.14 | P-type Mg(2+) transporter | Metal resistance/transport |
| 7.2.2.15 | P-type Ag(+) transporter | Metal resistance/transport |
| 7.2.2.16 | ABC-type ferric hydroxamate transporter | Metal resistance/transport |
| 7.2.2.17 | ABC-type ferric enterobactin transporter | Metal resistance/transport |
| 7.2.2.18 | ABC-type ferric citrate transporter | Metal resistance/transport |
| 7.2.2.19 | H(+)/K(+)-exchanging ATPase | Metal resistance/transport |
| 7.2.2.2 | ABC-type Cd(2+) transporter | Metal resistance/transport |
| 7.2.2.3 | P-type Na(+) transporter | Metal resistance/transport |
| 7.2.2.4 | ABC-type Na(+) transporter | Metal resistance/transport |
| 7.2.2.5 | ABC-type Mn(2+) transporter | Metal resistance/transport |
| 7.2.2.6 | P-type K(+) transporter | Metal resistance/transport |
| 7.2.2.7 | ABC-type Fe(3+) transporter | Metal resistance/transport |
| 7.2.2.8 | P-type Cu(+) transporter | Metal resistance/transport |
| 7.2.2.9 | P-type Cu(2+) transporter | Metal resistance/transport |
| 7.3.2.1 | ABC-type phosphate transporter | Metal resistance/transport |
| 7.3.2.2 | ABC-type phosphonate transporter | Metal resistance/transport |
| 7.3.2.3 | ABC-type sulfate transporter | Metal resistance/transport |
| 7.3.2.4 | ABC-type nitrate transporter | Metal resistance/transport |
| 7.3.2.5 | ABC-type molybdate transporter | Metal resistance/transport |
| 7.3.2.6 | ABC-type tungstate transporter | Metal resistance/transport |
| 7.3.2.7 | Arsenite-transporting ATPase | Metal resistance/transport |
